# Forest elephant movement and habitat use in a tropical forest-grassland mosaic in Gabon

**DOI:** 10.1371/journal.pone.0199387

**Published:** 2018-07-11

**Authors:** Emily C. Mills, John R. Poulsen, J. Michael Fay, Peter Morkel, Connie J. Clark, Amelia Meier, Christopher Beirne, Lee J. T. White

**Affiliations:** 1 Nicholas School of the Environment, Duke University, Durham, North Carolina, United States of America; 2 Agence Nationale des Parcs Nationaux, Batterie IV, Libreville, Gabon; 3 Independent Researcher, Karasburg, Namibia; 4 Institut de Recherche en Écologie Tropicale, Libreville, Gabon; 5 African Forest Ecology Group, School of Natural Sciences, University of Stirling, Scotland, United Kingdom; University of Tasmania, AUSTRALIA

## Abstract

Poaching of forest elephants (*Loxodonta cyclotis*) for ivory has decimated their populations in Central Africa. Studying elephant movement can provide insight into habitat and resource use to reveal where, when, and why they move and guide conservation efforts. We fitted 17 forest elephants with global positioning system (GPS) collars in 2015 and 2016 in the tropical forest-grassland mosaic of the Wonga Wongué Presidential Reserve (WW), Gabon. Using the location data, we quantified movement distances, home ranges, and habitat use to examine the environmental drivers of elephant movements and predict where elephants occur spatially and temporally. Forest elephants, on average, traveled 2,840 km annually and had home ranges of 713 km^2^, with males covering significantly larger home ranges than females. Forest elephants demonstrated both daily and seasonal movement patterns. Daily, they moved between forest and grassland at dawn and dusk. Seasonally, they spent proportionally more time in grassland than forest during the short-wet season when grasses recruit. Forest elephants also traveled faster during the short-wet season when fruit availability was greatest, likely reflecting long, direct movements to preferred fruiting tree species. Forest elephants tended to select areas with high tree and shrub density that afford cover and browse. When villages occurred in their home ranges elephants spent a disproportionate amount of time near them, particularly in the dry season, probably for access to agricultural crops and preferred habitat. Given the importance of the grassland habitat for elephants, maintenance of the forest-grassland matrix is a conservation priority in WW. Law enforcement, outreach, and education should focus on areas of potential human-elephant conflict near villages along the borders of the reserve. GPS-tracking should be extended into multi-use areas in the peripheries of protected areas to evaluate the effects of human disturbance on elephant movements and to maintain connectivity among elephant populations in Gabon.

## Introduction

Poaching of forest elephants, *Loxondonta cyclotis*, for ivory is decimating their populations [1]. Between 2002 and 2011, populations in Central Africa decreased by 62% and lost 30% of their geographical range due to global demand for ivory [2]. The densely forested country of Gabon is one of the last strongholds of forest elephants, housing about half of the world’s surviving forest elephants [2,3]. But poaching has taken a toll on Gabon’s elephant populations–approximately 25,000 elephants were lost from the Minkébé National Park in a decade [4].

With forest elephants under intense poaching pressure, information on their habitat use, movements, and ecology is necessary to maximize the effectiveness of conservation efforts. Movement ecology can provide insights into species’ resource requirements—food, water, and space—and elucidate temporal and spatial patterns of habitat use. Using global positioning systems (GPS) technology, animal movement studies have guided management strategies for a variety of terrestrial species [5], including identifying critical habitat for endangered species [6,7] and understanding resource-driven animal migrations [8]. Tracking movement can also detect changes to animal behavior in landscapes disturbed by urban development, extractive land use, and recreation, with implications for managing these activities [6,9,10]. Movement data from wide-ranging animals, such as wolves and elephants, have provided the empirical basis for identifying and securing movement corridors, particularly between large, protected wilderness areas [11,12].

Most current knowledge of the movement ecology of elephants comes from studies of savanna elephants, *Loxidonta africana*, in southern [13–15] and eastern Africa [16–18]. Vegetation (tree cover and food resources) and water limitation during the dry seasons are the main drivers of savanna elephant movements [13–15,17,18]. Savanna elephants are most active at night when temperatures fall [13], avoiding areas of high human density outside of protected areas [18]. In areas of low human density, elephants tend to avoid settlements during the daytime, with males more likely to approach villages than females at night [15].

By contrast, studies of forest elephant movement are rare because of the difficulty of collaring elephants in dense forests and, until recently, the challenge of reliably transmitting GPS signals through the canopy. Only five studies to date have used GPS collars to monitor forest elephant movements: a preliminary study successfully tracked one female elephant [19] and two additional studies derived descriptive metrics of home ranges and activity patterns [20,21]. Another study used random walk models to characterize forest elephant movements [22]. The largest study, consisting of 28 forest elephants, is the only study to use GPS collar data to model determinants of elephant movements, finding that unprotected roads acted as major barriers to movement [23]. These studies demonstrate that forest elephant movements are constrained by human disturbance except in the rare areas where elephants are safe from poaching [20], and that home range size varies greatly across both protected and human-use zones. Much remains to be learned regarding the relative effects of ecological and anthropogenic drivers of forest elephant movements and how they change across seasons, sites, and levels of protection.

In 2015 and 2016, the Gabon Parks Agency (ANPN) collared 17 forest elephants in the Wonga Wongué Presidential Reserve (WW) to assess their movements in relation to environmental and anthropogenic variables. Situated on the western coast of Gabon, WW consists of a forest-grassland matrix and is surrounded by 59 villages. Using hourly GPS locations, we characterize movements, estimate home ranges, and model the drivers of movements to test the following hypotheses:

1. Male forest elephants travel farther and cover larger home ranges than females because of their greater exploratory movements and lower risk avoidance;
2. Forest elephants spend more time in forest than grassland during the dry season and daytime to avoid exposure to sunlight, high temperatures, and poaching;
3. Forest elephants avoid roads and villages because of poaching threats, with females demonstrating greater avoidance than males;
4. During the dry season when water is limiting, forest elephants stay relatively close to permanent water sources, particularly streams and lakes.

In this paper, we improve our understanding of the movement ecology of forest elephants in a relatively well-protected reserve in Gabon. Based on our results, we propose management strategies for the conservation of forest elephants.

## Materials and methods

### Forest elephant data

In October 2015, PM led a field team to fit 12 forest elephants with GPS collars in WW and collared five additional elephants in April 2016 (S1 Table). The Gabon Parks Agency (ANPN) and Center for Scientific Research and Technology (CENAREST) reviewed and approved all darting and collaring methods, including the research ethics, before the work was conducted. The Duke Institutional Animal Care and Use Committee (IACUC) also reviewed the research, but no approval number was obtained because Duke’s role in the research began after the elephants were collared by ANPN. All the elephants were darted with a standard dose of 5mg of etorphine hydrochloride (Captivon). Sterile water was added to the etorphine to achieve a dart volume of 2 ml. The darts had a Cap-Chur 2cc aluminium barrel, neoprene plunger, and 1-3cc internal charge. We employed a Joubert Capture Equipment 45 mm x 3.5 mm barbed stainless steel needle and plastic flight. To fire the darts, we used a Dan-inject JM Special dart gun, powered by compressed carbon dioxide, with a 13 mm smooth barrel and a red-dot sight.

A standard dose of 5 mg was used for all adult elephants. When darting elephants in the forest with low visibility, the sex and age class of the animal are difficult to ascertain. Thus, we selected a dose that would stop an adult male, but not overdose an adult female. If darted in a good muscle mass, the elephant was usually recumbent within seven minutes. If the animal was lying on its sternum, the field team would push it onto its side. Most elephants were immediately given 10 mg butorphanol intravenously to improve ventilation and blood oxygenation. We carefully observed blood oxygenation and respiratory rate and depth and monitored heart rate with a pulse oximeter. Care was taken to ensure that airflow through the trunk was unobstructed and the trunk was held above pooled water if necessary. The dart wound was treated with 4 ml of 100 mg/ml oxytetracycline, and after fitting the collar, the elephants were woken up with 12 mg diprenorphine (Activon) and 50 mg of naltrexone (Trexonil) given intravenously.

The focal elephants consisted of seven females and 10 males, with age categories ranging from juveniles to old adults (S1 Table). The GPS collars transmitted coordinates hourly, with a successful transmission rate of approximately 90%. In this study, we use data from November 4, 2015 to March 4, 2017, consisting of 158,755 locations.

### Study area

**Fig 1:**
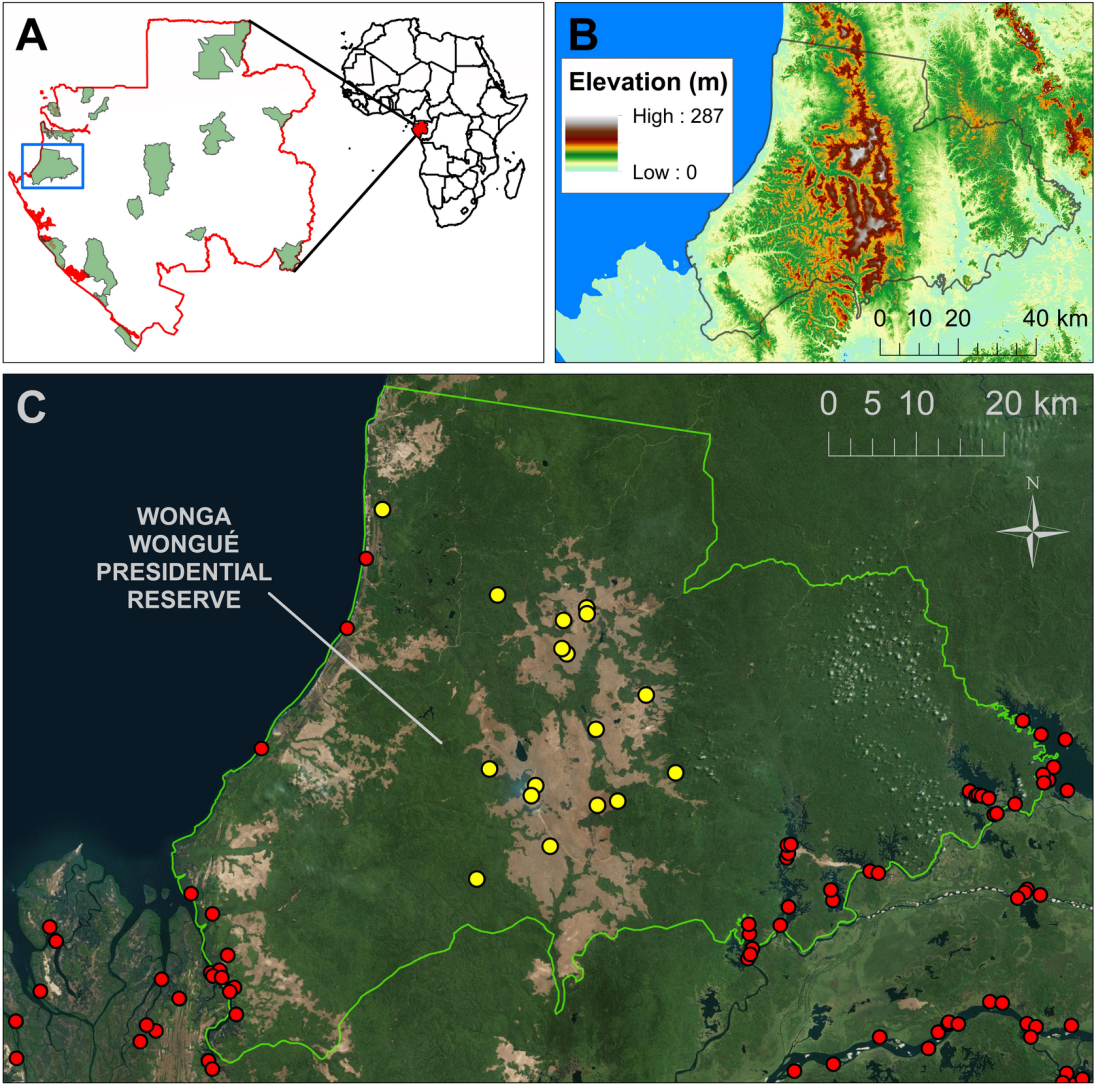
Study area. (A) Location of Wonga Wongué Presidential Reserve in Gabon. (B) Elevation map of WW: elevation is low except for the raised central grassland. (C) Location of collaring sites (yellow points) for 17 forest elephants in Wonga Wongué Presidential Reserve. Green land cover is forest, brown land cover is grassland, and small white dots indicate clouds. Human population is low in the reserve, but 59 villages (red points) are located within 10 km of its border.

Within WW (425,000 ha), habitats vary from white sand beaches and mangrove wetlands on the Atlantic coast to a mosaic of open grasslands and tropical forest in the interior (Fig 1). Grassland covers 15% of the reserve, with the central grassland (64,000 ha) forming a tropical forest-grassland mosaic. ANPN conducts prescribed burns annually during the long-dry season to maintain the grasslands, which would otherwise be overtaken by forest encroachment. Human population density is 0.2 people km^-2^, but 59 villages are located within 10 km of the reserve’s southwest and southeast borders (Fig 1). Elephants are free-roaming in the area and the reserve is well protected–no elephants had been poached within WW from 2014 to 2016 (pers. comm. David Fine).

### Land cover classification

To create a land cover map and examine landscape characteristics such as vegetation and wetness, we obtained Landsat 8 OLI/TIRS imagery (30 m resolution; S2A Table) from the U.S. Geological Survey (USGS) [24]. We used four images to cover the study area, one pair from the long-dry season and one pair from the long-wet season (S2B Table). All remote sensing analyses were performed in ENVI version 5.3 [25], unless otherwise specified. To ground truth the remote sensing imagery, ECM collected habitat and vegetation data in WW from May-July 2016, characterizing 220 recent elephant locations (S1 Fig; S3A Table). We created a thematic land cover map for the study area using the pair of images taken during the dry season because the ground truth data was collected during the dry season. To distinguish land cover categories, we employed maximum likelihood supervised classification using regions of interest (ROIs) created from the ground truth points as a training sample (S3B Table). We performed an accuracy assessment on the final classification using randomly generated ground truth points and Google Earth to evaluate the performance of the supervised classification technique. We excluded unclassified and rare pixels (those containing sand, chalk, or other habitat) because we were primarily interested in correctly classifying forest vs. grassland types. For both dry and wet season images, we created spectral enhancement bands for enhanced vegetation index (EVI), using coefficients adopted from the MODIS-EVI algorithm [24], and Tasseled Cap Transformation (TCT) bands for Brightness, Greenness, and Wetness, using coefficients derived by Baig *et al*. [26]. We used these rasters as habitat covariates in habitat modeling (see *Drivers of forest elephant movements* methods). Detailed classification methods are presented in S1 Text.

### Forest elephant movements and home ranges

To characterize elephant movements, we mapped and analyzed track distances across temporal variables (time of day and season) and elephant sex. We used generalized linear mixed models (GLMM) to evaluate whether these variables influence hourly rates and daily distances of movement (see *Model selection details* below). For hourly movement rates, we discarded coordinates recorded more than one hour after the previous coordinate, and treated individual elephant and hour as nested random effects. For daily movements, we only used days with at least 23 recorded locations, and treated individual elephant and day as random effects. Because of our small sample size (17 elephants), we tested for differences in total distance traveled by sex and season by bootstrapping the data 1,000 times with replacement and calculating 95% confidence intervals. To examine the spatial extent of individual forest elephant movements across time and to visualize high-use habitat areas, we generated home range polygons (see below) and again used bootstrapping to test for differences in home range area across sex and season.

We defined ‘home range’ as the area used by a given individual during its routine activities (including, but not limited to, foraging, mating, and predator avoidance) [27] over a specified period of time (in this case—annual or seasonal). We employed two different methods to estimate and visualize home ranges: minimum convex polygon (MCP) and kernel utilization distribution (KUD) [18,28]. We produced 100% and 95% MCPs as well as 95% and 50% KUD home ranges (using the reference bandwidth for the smoothing parameter, *h*; see [29] for equations). The MCP and KUD analyses were conducted in the *adehabitat* package in R version 3.3.1 [29,30]. The minimum bounding geometry and kernel density tools in ArcMap version 10.4.1 [31] were used for mapping MCP and KUD home ranges.

### Forest elephant habitat use

**Fig 2:**
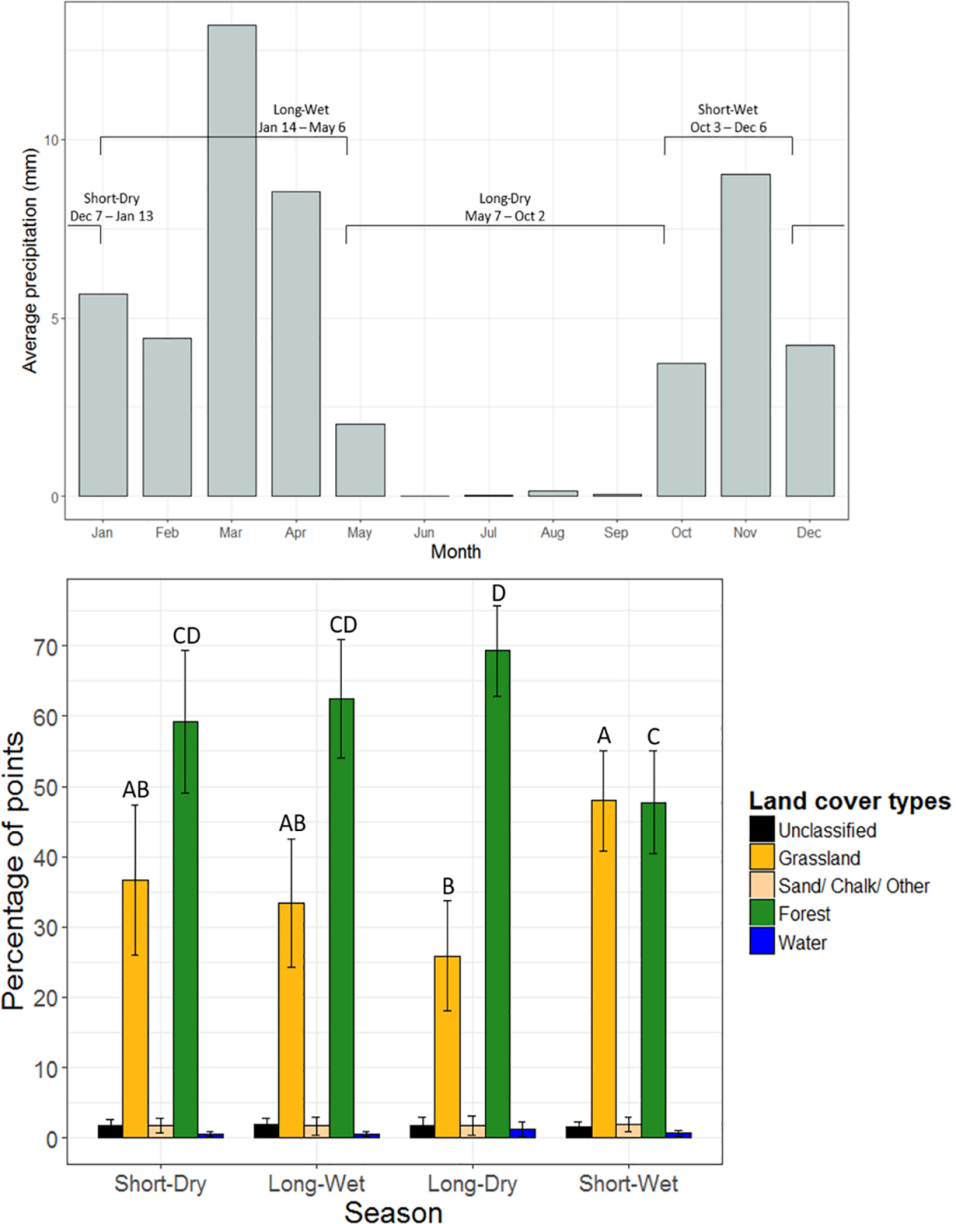
Forest elephant habitat use by season. (*Above*) Average precipitation (mm) by month, from which seasons were defined as the short-wet season (Oct. 3 –Dec. 6), short-dry season (Dec. 7 –Jan. 13), long-wet season (Jan. 14 –May 6), and long-dry season (May 7 –Oct. 2). Note that depicting rainfall by month fails to show the daily variation in precipitation that determines the seasons. (*Below*) Percentage of elephant locations in each land cover type by season. Pairwise comparisons with different letters indicate significant differences in proportion of use by elephants across seasons within land cover type. Error bars represent 95% confidence intervals.

To examine patterns of forest elephant habitat use, we extracted the land cover type at each hourly GPS location of the collared elephants. Time of day was split into four equal categories, using breaks at the approximate sunrise and sunset times: 0:00–5:59, 6:00–11:59, 12:00–17:59, and 18:00–23:59. We defined seasons using precipitation data from the Tropical Rainfall Measuring Mission (TRMM) from November 2015 to November 2016, with dry seasons characterized by < 60mm total rainfall as defined by the Koppen climate classification. The long-dry season (May 7 –Oct. 2) had no single rain event > 10 mm, the short-dry season (Dec. 7 –Jan. 13) had no single rain event > 20 mm, and the remaining two periods were designated as the long- (Jan. 14 –May 6) or short- (Oct. 3 –Dec. 6) wet seasons based on their duration (Fig 2). We used one-way ANOVA with *post-hoc* pairwise comparisons (Bonferroni correction) to analyze differences in land cover types at elephant locations across seasons and times of day.

### Drivers of forest elephant movements

To determine the main drivers of forest elephant movements in WW, we first assembled 11 *a priori* candidate variables hypothesized to influence elephant movement (S4 Table). Variables included distance and slope rasters, which we developed using ArcMap and original spatial data provided by ANPN. We also included spectral enhancement bands (as described above in *Land cover classification* methods) and created a raster as a measure of forest openness, by estimating the proportion of pixels classified as forest within a 90 m circular neighborhood.

We then built a habitat model using a generalized linear mixed model (GLMM) with a presence/pseudoabsence design [32]. Presence points were GPS locations of elephants and pseudoabsence points–a representative sample of available habitat not used within home ranges–were randomly selected points within the 100% MCP home range of each elephant. Pseudoabsence points could be randomly selected from the same pixel (900 m^2^) as other pseudoabsence points, but a 50 m buffer was maintained around presence points to prevent contamination of presence and pseudoabsence points (within the same 30 m cell). We thinned presence points to one point per day to minimize temporal and spatial interdependence of consecutive points [33] and generated a single pseudo-absence point per day.

We modeled wet and dry seasons separately to account for seasonal differences in availability of resources, such as grass, fruiting trees, and precipitation. We used mixed-effects logistic regression to model elephant presence for dry and wet seasons, with candidate variables treated as fixed effects and elephant identity treated as a random effect to account for lack of independence of points and unequal sample sizes among individuals [18]. To avoid flawed inference arising from multicollinearity, we evaluated correlations between all candidate variables (n = 11), removing one of the variables from any highly correlated pair (where *r* > 0.7). This resulted in a final set of 6 candidate variables: distance to nearest road (km), distance to the nearest village (km), distance to the nearest stream (km), slope (m), Enhanced Vegetation Index (EVI), and sex. Continuous covariates were standardized as z-scores to facilitate comparison of effect sizes. Given that all remaining covariates were uncorrelated, we tested all combinations of covariates. This resulted in a suite of 64 candidate models for each season (see *Model selection details* below). For each best-supported seasonal habitat model, we also employed *k*-fold cross validation with a testing ratio of 20% to evaluate model performance [32].

#### Model selection details

We applied an information theoretic approach to model selection, [34] whereby candidate models were ranked using Akaike’s Information Criterion corrected for small sample size (AICc). For each season, we defined a ‘top model set’ which included all models with a ΔAIC ≤ 6 from the best supported model, after excluding any models of which a simpler nested version attained stronger support (following the ‘nesting rule’ of [35]). This approach avoids the issue of selecting and interpreting spurious covariates. Informative covariates are discussed in terms of their relative importance, defined here as the sum of Akaike weights (SW) across all of the ‘top-set’ models in which the covariate occurs, and absolute importance, defined here as the model averaged effect-size for each covariate. GLMMs were fitted in the *lme4* package in R [36,30].

## Results

### Land cover classification

The land cover map accurately classified the three most common land cover categories (forest, grassland, water) with 98.4% accuracy (Kappa = 0.977; S5 Table; S2 Fig). Mangroves and swamps were poorly distinguished from forest because of their restricted representation and small number of ground truth points.

### Forest elephant movements and home ranges

There was no support for sex differences in hourly movement rates (Table 1). Elephants traveled significantly faster in grassland than in any other land cover type (Table 1). Elephants moved faster near dawn (6:00–8:00) and dusk (17:00–20:00) than other time periods (S3 Fig), but hourly movement rates varied significantly with season (significant interaction between time of day and season, Table 1). Similarly, female and male elephants did not exhibit significant differences in daily movement rates, although elephants traveled significantly greater distances during the wet season than the dry season (Table 1; S4 Fig).

**Table 1:** Hourly and daily movement rates statistics.

**Hourly Distance Traveled (km)**					
**Fixed Effects**	***SW***	**Fixed Effect Contrasts**	**Coef**	**95% CI**
Intercept		-	0.307	[0.284, 0.330]
Land Cover	1.00	Forest	-0.064	[-0.068, -0.060]
		Sand/Chalk/Other	-0.077	[-0.090, -0.064]
		Unclassified	-0.059	[-0.071, -0.046]
		Water	-0.060	[-0.079, -0.041]
Time of day (TOD)	1.00	06:00–11:59	0.049	[0.026, 0.071]
		12:00–17:59	0.059	[0.037, 0.081]
		18:00–24:00	0.045	[0.023, 0.067]
Season	1.00	Wet	0.053	[0.045, 0.060]
Season * Time of day	1.00	Season (Wet): TOD (06:00–11:59)	-0.019	[-0.029, -0.009]
		Season (Wet): TOD (12:00–17:59)	-0.043	[-0.053, -0.033]
		Season (Wet): TOD (18:00–24:00)	-0.007	[-0.016, 0.003]
Sex	0.00	-	-	-
**Random Effects**		** **	**SD**	
Elephant ID			0.001	
Hour			0.005	
**Daily Distance Traveled (km)**					
**Fixed Effects**	***SW***	**Fixed Effect Contrasts**	**Coef**	**CI**
Intercept	1.00		6.888	[6.392, 7.387]
Season	1.00	Wet	1.108	[0.800, 1.415]
Sex	0.00	-	-	-
**Random Effects**		** **	**SD**	
Elephant ID			0.836	
Day			1.703	

Sums of weight (SW), coefficients (Coef), and confidence intervals (CI), from the model averaged top model set GLMM of hourly (*above*) and daily (*below*) movement rates of elephants. Intercepts relate to grassland, dry season, and the 00:00–05:59 time period for the hourly movement analysis, and dry season for the daily movement analysis. Contrasts are only shown for fixed effects that have some support under model selection (SW > 0). For full model selection output see S6 Table.

**Fig 3:**
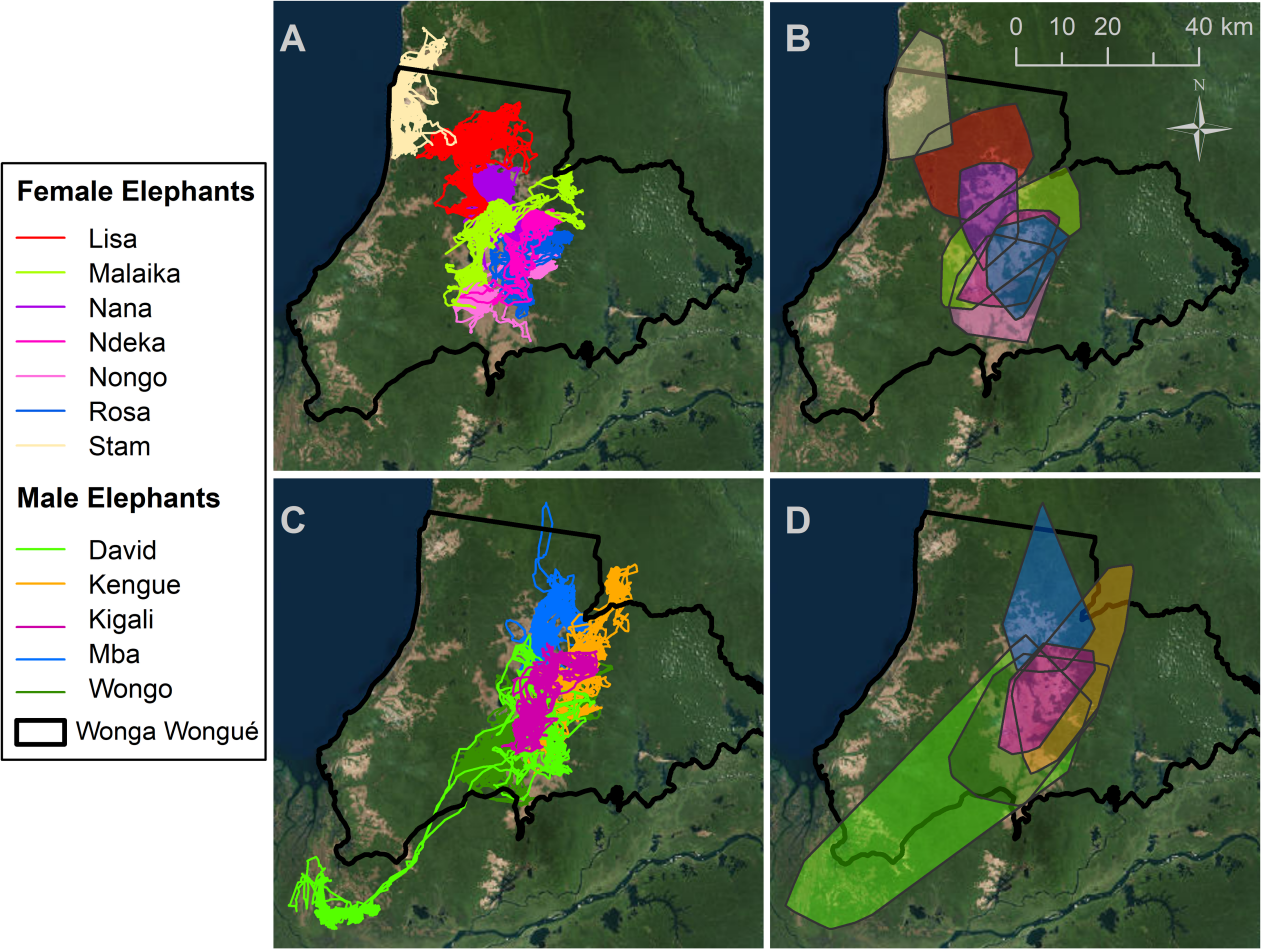
Forest elephant tracks and home ranges. Movement tracks (A and C) and 100% MCP home ranges (B and D) for female elephants (*top row*) and a subset of male elephants (*bottom row*). Female elephants tended to stay closer to the central grassland, whereas half of the males traveled up to 110 km from the central grassland to core home range areas in the reserve periphery.

Elephants traveled an average of eight km per day and 3,444 km over the study period (Fig 3; S7 Table). The 12 elephants with at least one full year of location data traveled 2,840 km annually on average. Total distance traveled by elephants did not differ significantly by sex or season (Table 2). The average 100% MCP home range, which has been most commonly used in previous studies, was 713 km^2^ (Table 2; S8 Table). Using 100% MCP, male elephants covered significantly larger home ranges than female elephants, but home ranges did not differ significantly between wet and dry seasons (Fig 3; Table 2; S9 Table; S5 Fig). The size of core areas, represented by 50% KUD areas, did not differ between sexes (Table 2; S10 Table).

**Table 2:** Distance and home range statistics.

Metric	Variable	Mean	95% CI	Variable	Mean	95% CI
Distance, km	Female	2326	[2067, 2573]	Male	2395	[2288, 2501]
	Wet	1864	[1569, 2157]	Dry	1625	[1499, 1747]
Home Range, km^2^						
50% KUD	Female	44.4	[29.0, 59.7]	Male	105.1	[53.0, 156.1]
**95% KUD**	Female	211.9	[149.2, 273.3]	Male	654.3	[339.1, 973.9]
**95% MCP**	Female	254.1	[179.4, 332.2]	Male	766.0	[416.9, 1107.2]
**100% MCP**	Female	353.9	[278.6, 430.5]	Male	965.0	[587.7, 1345.2]
100% MCP	Wet	538.4	[297.9, 786.6]	Dry	526.5	[309.7, 756.6]

Bootstrapped 95% confidence intervals (CI) for total distance traveled and various home range metrics. Total distance traveled was calculated over the 10 months for which data was available for all elephants (May 2016-March 2017). Bolded metric names denote statistical significance between variables based on non-overlapping 95% CIs. See supporting information for summary distance metrics (S7 Table) and home range metrics (S8, S9 and S10 Tables) by individual elephant.

### Forest elephant habitat use

Forest elephants spent 62% of their time in forests and 33% in grasslands (males: 78% forest, 20% grassland; females: 68% forest, 30% grassland; Fig 2). Use of forest varied among seasons, with significantly more locations in forest during the long-dry than the short-wet season (ANOVA: *F*_*3*,*64*_ = 4.2, *p* = 0.009; Fig 2). During daytime hours, when sun exposure and temperatures are highest, elephants spent significantly more time in forest than grassland (ANOVA: *F*_*3*,*64*_ = 24.4, *p* < 0.001; S6 Fig). Peak usage of forest occurred between 6:00–12:00, and peak usage of grassland occurred between 18:00–24:00 (S6 Fig).

### Drivers of forest elephant movements

During the dry season, there was full support for distance to villages and EVI in predicting forest elephant habitat use (Table 3), whereas distance to streams and slope had weaker support. There was no support for distance to roads or sex in influencing elephant habitat use (Table 3). Distance to village most strongly predicted elephant presence, with twice the effect size of EVI. Elephant presence decreased with increasing distance from the village, whereas EVI, distance to stream, and slope had weaker, positive effects on elephant presence.

**Table 3:** Dry and wet season habitat models.

	Dry Season			Wet Season		
**Fixed Effects**	**SW**	**Coef**	***95% CI***	**SW**	**Coef**	***95% CI***
Intercept	-	0.011	[0.103,0.124]	-	0.016	[-0.046, 0.078]
Enhanced Vegetation Index	1.00	0.130	[0.080, 0.181]	1.00	0.223	[0.171, 0.275]
Distance to village	1.00	-0.262	[-0.346, -0.177]	0.85	-0.071	[-0.158, 0.016]
Distance to stream	0.87	0.052	[-0.008, 0.112]	1.00	0.184	[0.136, 0.232]
Slope	0.70	0.033	[-0.025, 0.090]	-	-	-
Distance to Roads	0.00	-	-	1.00	-0.090	[-0.136, -0.041]
Sex	0.00	-	-	-	-	-
**Random Effects**	**SD**			**SD**		
Elephant ID	0.219			0.220		

Sums of weight (SW), coefficients/effects size (Coef), and confidence intervals (CI) from the model averaged top model set GLMMs of elephant presence in dry and wet seasons. Coefficients are model averaged log-odds ratios. Using *k*-fold cross validation to assess model performance, the mean model accuracy for the dry season was 53.9% and for the wet season was 55.4%. For full model selection output see S11 and S12 Tables.

During the wet season, there was full support for EVI, distance to streams, and distance to roads in predicting forest elephant habitat use, and weaker support for distance to village (Table 3). There was no support for slope or sex. EVI most strongly influenced habitat use, with the probability of elephant presence increasing 25% with each one-unit increase in standardized EVI. The next strongest effect was distance to stream, which positively affected elephant presence. Distance to road and distance to village had weaker, negative effects on elephant presence.

## Discussion

Our study highlights distinct differences in temporal and seasonal use of habitat by forest elephants in a forest-grassland matrix. Forest elephants spend most of their time in forest, but selectively use grassland during nighttime hours and during the short-wet season, when young grass shoots provide browse. Grassland habitat is likely important for elephant resource use and social interactions, like the use of bais (forest clearings) for forest elephant social gatherings [37,38]. In the Wonga Wongué Presidential Reserve, whether environmental factors such as food, water, and habitat drive elephant movements more than anthropogenic disturbance was dependent on the season—elephants come into closer contact with villages in the dry season compared to the wet season. Elephants are free-roaming in the area and the reserve is well protected with low rates of poaching compared to other sites (e.g. [4]). Whereas previous studies of forest elephant movement focused on the importance of unprotected habitat or roads, we demonstrate that environmental variables can also strongly influence elephant movements where they are protected. Even so, forest elephants spent disproportionate time near villages in their home ranges, particularly in the dry season, underscoring that conservation efforts should focus on areas of potential human-elephant conflict along the populated peripheries of the reserve.

Despite our first hypothesis that male forest elephants would travel farther than females, both sexes travelled on average 7–8 km daily and 2,840 km annually. Peak elephant movements occurred around dawn and dusk, likely representing movements from grassland to forest in the morning and from forest to grassland in the evening. While this finding concurs with activity patterns of savanna elephants [13], it contrasts with the diurnal activity patterns of a female elephant in Congo that was most active between 12:00–21:00, with a peak at 15:00 [19]. Forest cover in closed canopy forest may protect elephants from heat [19], whereas elephants in a forest-grassland mosaic likely adapt their activity patterns to use the open grassland when temperatures are cooler. Whereas male and female elephants did not differ in total distances moved, they differed significantly in home range sizes. The average 100% MCP home range area for WW forest elephants was 713 km^2^, with males averaging 965 km^2^ and females averaging 354 km^2^—greater and less, respectively, than the average home range of 546.8 km^2^ across six sites in Central Africa [23]. These differences are consistent with male elephants using larger home ranges than females because they engage in exploratory movements outside core areas of habitat use—even outside park boundaries—to find forage and mates. Males are often solitary and have more fluid social interactions unconstrained by offspring [39], whereas females are more likely to be in small family groups with dependent offspring. Females constrained by offspring may behave more cautiously, remaining in known areas with reliable resources where it may also be easier to protect their young from predation.

Other studies of forest elephants have found that home ranges vary widely across sites. A female elephant in Ndoki National Park had an MCP home range of 2,226 km^2^ [23]; whereas three female elephants in an oil concession in southwest Gabon covered an average home range of 212 km^2^, perhaps constrained by human presence [20]. Home range sizes vary strongly across protected areas in Gabon, from an average MCP of 75.6 km^2^ in Loango National Park to 568.2 km^2^ in Minkébé National Park and 623.2 km^2^ in Ivindo National Park [23]. Six females in Loango National Park had an average 95% kernel home range of only 51.7 km^2^ [21]. From this small number of studies, it appears that apart from Loango, where home ranges are geographically constrained by the coastline and a large lagoon in the north of the park, forest elephants across Gabon have average home range areas of 500 to 600 km^2^. Consistent with elephants in Loango [21], females in WW maintained more separated home ranges than males. The variation in home range areas across sites underscores the need for detailed studies of elephant habitat to determine how fine scale differences in land use history, geology, soils, nutrient/mineral availability, and rainfall affect habitat quality, elephant resource use, and movement.

Consistent with our second prediction that forest elephants would spend more time in the forest than grassland habitat during the daytime and dry season, we found strong evidence for seasonal shifts in activity patterns and habitat use. Overall, forest elephants spend most of their time in forests, with approximately one-third of their time in grassland. During the dry season, elephants occur in the forests adjacent to the central grassland, but rarely venture into the grassland. Use of grassland habitat only surpassed forest in the short-wet season, when elephants congregate in the central grassland to browse on young grass following prescribed burns. Studies on savanna elephants have similarly shown that elephants select habitats with greater tree cover in the dry season, but not the wet season [17,40]. Reduced distances moved during the dry season could reflect the aggregation of water and fruit resources, such that: 1. Elephants stay near perennial water sources during times of relative water scarcity; and/or 2. Elephants stay near patches of *Sacoglottis gabonensis* trees, which supply a keystone fruit [41,42] and are abundant in WW during the long-dry season. By comparison, the greater hourly and daily distances moved in the wet season are likely a result of the onset of new grass growth in grasslands and fruiting of most tree species in the forest [43]. Elephants likely move greater distances as they travel between forest and grassland and as they make long direct movements to less common preferred fruiting species [44].

Elephants tend to visit grasslands at night, moving into forest during the daytime for protection from the sun and high temperatures in the grassland. Nighttime use of grasslands in WW is consistent with research on forest clearings: 79% of visits to the Dzanga-Sangha Bai in Central African Republic occurred at night [37]. Daytime use of forest habitat could also be an evolutionary adaptation to avoid predation or hunting. Relatively low rates of poaching in WW compared to other sites (e.g. [4]) over the last few years, however, suggests that environmental factors may play a stronger role than anthropogenic factors in driving elephant movements between forest and grassland.

Contrary to our third hypothesis that elephants would avoid villages, we found that elephants tended to spend time near villages in their home ranges, particularly in the dry season. Elephants may be attracted to secondary habitat and transitional areas near villages cleared for agriculture, logging, and road building [45]. The attraction of villages was particularly strong in the dry season, when fewer tree species produce fruits and new leaves. Although we did not detect a significant difference between sexes in average distance from villages, three male elephants noticeably ventured within 5 km of villages at the edges of the reserve (S7 Fig). Villagers from Gongoué, on the west coast of WW, observed a collared male elephant raiding their crops. As mentioned above, female elephants may be intrinsically more cautious because of offspring, whereas young, subordinate males may be more likely to raid crops or abandoned fields and lose fear of people, causing damage and conflict. Monitoring the distance of elephants from villages is important to assess the severity of crop raiding in the periphery of WW, to pinpoint target areas for conflict mitigation, and perhaps to identify problem elephants that need to be removed or relocated [46,47]. Repeated crop raiding could prompt villagers to kill elephants in retaliation. In February 2017, poachers killed a collared male elephant 5 km from a village southwest of the reserve. It is unknown whether the incident was motivated by crop raiding or the ivory trade, but bullets recovered from the carcass and the removal of tusks suggest the latter.

Similar to their attraction to villages, elephant presence increased near roads during the wet season and was unaffected by roads during the dry season. Although forest elephants avoid unprotected roads in areas where poaching and human disturbance is high [23,48], secondary or abandoned roads with little traffic may act as convenient movement routes for elephants. In a protected oil concession in Gabon, elephants occurred just 516 m from roads on average [20]. In WW, elephants tended to be closer to roads during the wet season, in contrast to our hypothesis that elephants would avoid roads. Male and female elephants crossed roads frequently, with males averaging 503 crossings and females averaging 342 crossings over the study period, although they crossed roads at significantly higher speeds than non-crossing movements (e.g., [23]).

Consistent with our final hypothesis, during the wet season, presence of elephants increased at greater distances from perennial water sources. This result is consistent with elephants moving farther during the wet season (>1 km per day) than in the dry season. The greater abundance of small, temporary water sources and decreased water limitation likely allows elephants to move farther away from permanent water supplies.

## Recommendations

Our study provides insights into the habitat use and behavior of forest elephants in a forest-grassland mosaic, but there is still a great deal to be learned about the movement ecology of forest elephants to effectively conserve their populations. Even with 17 GPS-collared elephants—the second-largest study of forest elephant movement—our sample size is low and additional tracking of elephants is necessary to reach robust conclusions regarding their movement, home range, and habitat use through space and time.

Forest-grassland mosaics pose some unique challenges for management of elephant populations, but protecting elephants from poaching is still the key to their conservation. Unlike the arid savannas of southern and eastern Africa where water management is a primary concern, in humid tropical forest the chief issues are maintaining grassland openness, promoting grass turnover, and preserving fruiting tree phenology and diversity. Prescribed burns are an important tool for maintaining grasslands that serve as sources of food resources and social interactions. The interior of WW is currently well protected from poachers, but conflict with humans could threaten elephants along its boundaries. Conservation monitoring and law enforcement should be focused in the periphery zone, especially near villages where elephants might raid crops. Future GPS-tracking efforts should focus on elephants, particularly males, in the periphery of protected areas to evaluate their interactions with villages and to warn park management, e.g. via SMS text alerts, when elephants approach plantations [49]. Raising awareness of local communities about the GPS monitoring program and elephant ecology, and investing in methods to prevent crop raiding could further mitigate threats.

Comparing elephant habitat use inside, outside, and near the borders of protected areas will help to assess the effects of anthropogenic disturbance on elephant movements. As economic development progresses—particularly with the growth of extractive industries—natural landscapes are fragmented, confining large, mobile species to islands of suitable habitat. Maintaining connectivity between protected areas will enhance the capacity of Gabon’s protected area network to conserve forest elephant populations and ensure they remain a long-term component of Central African forests.

## Supporting information

S1 Table Seventeen forest elephants collared in Wonga Wongué Presidential Reserve. (PDF)

S2 Table Landsat information. (PDF)

S3 Table Ground truth points and regions of interest. (PDF)

S4 Table Environmental variables of interest included in habitat modeling. (PDF)

S5 Table Confusion matrices in pixels and percentages. (PDF)

S6 Table Full model selection outputs for hourly and daily movements. (PDF)

S7 Table Total track distance summaries for 17 GPS-collared forest elephants in WW. (PDF)

S8 Table Area of MCP home ranges. (PDF)

S9 Table 100% MCP home range areas for WW by elephant and season. (PDF)

S10 Table Area of KUD home ranges. (PDF)

S11 Table Full model selection output for the factors influencing elephant movement in the dry season. (PDF)

S12 Table Full model selection output for the factors influencing elephant movement in the wet season. (PDF)

S1 Fig GPS locations and ground truth points. (PDF)

S2 Fig Thematic land cover map for the study area. (PDF)

S3 Fig Boxplots showing distances moved between consecutive GPS points for all hours of the day. (PDF)

S4 Fig Boxplots showing distributions of distance between consecutive GPS points by month. (PDF)

S5 Fig 95% MCP and 95% KUD home ranges for forest elephants in WW. (PDF)

S6 Fig Percentage of elephant locations in each land cover type by time of day. (PDF)

S7 Fig Distance to nearest villages by month (*above*) and by elephant (*below*). (PDF)

S1 Text Land cover classification: Detailed protocol. (PDF)
