# Supplementary material for: Forest elephant movement and habitat use in a tropical forest-grassland mosaic in Gabon
Source: PLoS One. 2018 Jul 11;13(7):e0199387. doi: 10.1371/journal.pone.0199387 (PMC6040693; doi:10.1371/journal.pone.0199387)
Supplement: S1 Table — (PDF) [file pone.0199387.s001.pdf]

S1 Table. Seventeen forest elephants collared in Wonga Wongué Presidential Reserve.

Elephant Name	ID Code	Sex	Date Collared	Date Start Hourly Pings	Total GPS Points
Rosa	Duke01	F	10/23/2015	11/4/2015	10,759
Nana	Duke02	F	10/26/2016	11/4/2015	10,922
Lisa	Duke04	F	10/22/2015	11/4/2015	10,015
Ndeka	Duke06	F	10/26/2015	11/4/2015	10,875
Stam	Duke09	F	10/29/2016	11/4/2015	9,309
Nongo	Duke16	F	4/30/2016	05/12/2016	6,892
Malaika	Duke20	F	4/29/2016	05/12/2016	6,790
Kengue	Duke03	M	10/23/2015	11/4/2015	10,233
Mba	Duke05	M	10/25/2015	11/4/2015	10,467
Mambo	Duke07	M	10/24/2015	11/4/2015	10,668
Mboumba*	Duke08	M	10/25/2015	11/4/2015	10,543
David**	Duke10	M	10/24/2015	11/4/2015	10,241
Wongo	Duke11	M	10/28/2016	11/4/2015	10,876
Nze	Duke12	M	10/28/2016	11/4/2015	10,417
BraBrou	Duke19	M	4/30/2016	05/12/2016	6,630
Tonnerre	Duke31	M	4/29/2016	05/12/2016	6,489
Kigali	Duke32	M	4/30/2016	05/12/2016	6,629

*Mboumba’s GPS collar ceased transmission on February 12, 2017. He has not been relocated, suggesting potential collar failure.

**Poachers killed the elephant, David, whose last GPS transmission was on February 22, 2017. Field teams located the carcass with ivory and head removed, and the GPS collar in a nearby swamp.

Elephants GPS locations from November 4, 2015 to March 4, 2017 were analyzed. Table sorted by sex and ID code.
