# Supplementary material for: Forest elephant movement and habitat use in a tropical forest-grassland mosaic in Gabon
Source: PLoS One. 2018 Jul 11;13(7):e0199387. doi: 10.1371/journal.pone.0199387 (PMC6040693; doi:10.1371/journal.pone.0199387)
Supplement: S2 Table — (PDF) [file pone.0199387.s002.pdf]

S2 Table. Landsat information.

Table A. Landsat 8 OLI/TIRS Bands.

Band Number	Purpose	Wavelength (μm)	Spatial Resolution (m)
Band 1	Ultra Blue (coastal/aerosol)	0.43 - 0.45	30
Band 2	Blue	0.45 - 0.51	30
Band 3	Green	0.53 - 0.59	30
Band 4	Red	0.64 - 0.67	30
Band 5	Near Infrared (NIR)	0.85 - 0.88	30
Band 6	Shortwave Infrared (SWIR) 1	1.57 - 1.65	30
Band 7	Shortwave Infrared (SWIR) 2	2.11 - 2.29	30
Band 8	Panchromatic	0.50 - 0.68	15
Band 9	Cirrus	1.36 - 1.38	30
Band 10	Thermal Infrared (TIRS) 1	10.60 - 11.19	100* (30)
Band 11	Thermal Infrared (TIRS) 2	11.50 - 12.51	100* (30)

*Thermal bands resampled to 30m

Table B. Landsat scene metadata.

Scene ID	Orientation	Date Taken	Time Taken	Product Level
LC81860602015179LGN00	Northern	2015-06-28	09:33:24	1T
LC81860612015179LGN00	Southern	2015-06-28	09:33:48	1T
LC81860602016070LGN00	Northern	2016-03-10	09:33:54	1T
LC81860612016070LGN00	Southern	2016-03-10	09:34:18	1T
