# Supplementary material for: Forest elephant movement and habitat use in a tropical forest-grassland mosaic in Gabon
Source: PLoS One. 2018 Jul 11;13(7):e0199387. doi: 10.1371/journal.pone.0199387 (PMC6040693; doi:10.1371/journal.pone.0199387)
Supplement: S3 Table — (PDF) [file pone.0199387.s003.pdf]

S3 Table. Ground truth points and regions of interest.

Table A. Description of ground truth points collected from June 6, 2016 to July 21, 2016.

Descriptive Statistics		Ecosystem Type	Number of points
Number of points	220	Forest	151
Number of individual elephants followed	8	Grassland	52
Average positional dilution of precision (PDOP)	1.63	Swamp	6
Average error (m)	8.27	Beach	4
Average canopy height (m)	17.34	Mangroves	4
Average canopy cover (%)	97.0	Transitional	3

Attributes collected included land cover type, canopy cover, understory density, tree height, and most abundant tree species.

Table B. Original classes of regions of interest (ROIs) and total training pixels per tile.

Class	Training Pixels (North)	Training Pixels (South)	Validation Points (Total)
Grassland	338	141	97
Forest	151	35	82
Water	137	105	79
Sand/ Chalk/ Other	24	25	-

Swamp and mangrove pixels were excluded from analysis due to poor separability from forest.
