# Supplementary material for: Forest elephant movement and habitat use in a tropical forest-grassland mosaic in Gabon
Source: PLoS One. 2018 Jul 11;13(7):e0199387. doi: 10.1371/journal.pone.0199387 (PMC6040693; doi:10.1371/journal.pone.0199387)
Supplement: S4 Table — (PDF) [file pone.0199387.s004.pdf]

S4 Table. Environmental variables of interest included in habitat modeling.

Category of Interest	Raster Layers
Human Influence	Distance to secondary roads (km)<br>Distance to villages (km)
Terrain characteristics	Elevation (m)<br>Slope (m)
Vegetation characteristics	Land Cover Type Classification<br>Enhanced Vegetation Index (EVI)<br>Tasselled Cap Transformation (TCT) Greenness
Openness/Exposure	Focal Forest<br>Tasselled Cap Transformation (TCT) Brightness
Water sources	Distance to streams (km)<br>Tasselled Cap Transformation (TCT) Wetness
