# Supplementary material for: Forest elephant movement and habitat use in a tropical forest-grassland mosaic in Gabon
Source: PLoS One. 2018 Jul 11;13(7):e0199387. doi: 10.1371/journal.pone.0199387 (PMC6040693; doi:10.1371/journal.pone.0199387)
Supplement: S5 Table — (PDF) [file pone.0199387.s005.pdf]

S5 Table. Confusion matrices in pixels and percentages.

	Ground Truth (Pixels)			
**Classification**	**1- Grassland**	**3- Forest**	**4- Water**	**Total**
**Grassland**	93	4	0	97
**Forest**	0	82	0	82
**Water**	0	0	78	78
**Total**	93	86	78	257

	Ground Truth (Percent)			
**Classification**	**Grassland**	**Forest**	**Water**	**Total**
**Grassland**	100.00%	4.65%	0.00%	37.74%
**Forest**	0.00%	95.35%	0.00%	31.91%
**Water**	0.00%	0.00%	100.00%	30.35%
**Total**	100.00%	100.00%	100.00%	100.00%

The three main land cover types of interest—grassland, forest, and water—assessed for classification accuracy. Matrix diagonals show correctly classified pixels.
