# Supplementary material for: Forest elephant movement and habitat use in a tropical forest-grassland mosaic in Gabon
Source: PLoS One. 2018 Jul 11;13(7):e0199387. doi: 10.1371/journal.pone.0199387 (PMC6040693; doi:10.1371/journal.pone.0199387)
Supplement: S6 Table — (PDF) [file pone.0199387.s006.pdf]

S6 Table. Full model selection outputs for hourly and daily movements

Table A. Full model selection output for the factors influencing hourly movement.

Where: ✓ = categorical terms included in the model; TOD = Time of day; df = Degrees of Freedom; AICc = Akaike's Information Criterion corrected for small sample size; W = model weight; SW = Sums of weight across all models in the top set; grey shaded area = all models in the top model set (ΔAICc < 6 and non-nested).

Intercept	Forest Type	Season	Sex	TOD * Season	df	AICc	ΔAICc	W	SW
0.31	✓	✓		✓	15	72458.2	0.00	0.69	1.00
0.30	✓	✓	✓	✓	16	72459.7	1.55	0.32	0.00
0.32	✓	✓			12	72545.1	86.96	0.00	0.00
0.31	✓	✓	✓		13	72546.7	88.51	0.00	0.00
0.35	✓	✓			9	72557.1	98.88	0.00	0.00
0.34	✓	✓	✓		10	72558.6	100.43	0.00	0.00
0.34	✓				11	72957.7	499.51	0.00	0.00
0.33	✓		✓		12	72959.2	501.01	0.00	0.00
0.37	✓				8	72969.6	511.41	0.00	0.00
0.36	✓		✓		9	72971.1	512.91	0.00	0.00
0.27		✓		✓	11	73423.2	965.02	0.00	0.00
0.26		✓	✓	✓	12	73425.1	966.89	0.00	0.00
0.28		✓			8	73524.0	1065.86	0.00	0.00
0.27		✓	✓		9	73525.9	1067.73	0.00	0.00
0.30		✓			5	73537.7	1079.51	0.00	0.00
0.30		✓	✓		6	73539.6	1081.37	0.00	0.00
0.30					7	74123.9	1665.70	0.00	0.00
0.29			✓		8	74125.7	1667.56	0.00	0.00
0.32					4	74136.9	1678.73	0.00	0.00
0.32			✓		5	74138.8	1680.58	0.00	0.00

Table B. Full model selection output for the factors influencing daily movement.

Intercept	Season	Sex	df	AICc	ΔAICc	W
6.89	✓		5	16927.2	0.00	0.59
6.57	✓	✓	6	16928.0	0.76	0.41
7.47			4	16970.3	43.11	0.00
7.16		✓	5	16971.1	43.91	0.00
