# Supplementary material for: Forest elephant movement and habitat use in a tropical forest-grassland mosaic in Gabon
Source: PLoS One. 2018 Jul 11;13(7):e0199387. doi: 10.1371/journal.pone.0199387 (PMC6040693; doi:10.1371/journal.pone.0199387)
Supplement: S7 Table — (PDF) [file pone.0199387.s007.pdf]

S7 Table. Total track distance summaries for 17 GPS-collared forest elephants in WW.

Elephant	Sex	Total distance (km)	Total distance-10 month (km)	Average distance per day (km)	Average distance per hour (m)
Malaika*	F	2,219	2,219	7.50	312
Nongo*	F	2,754	2,754	9.30	388
Rosa	F	2,883	1,771	5.93	247
Ndeka	F	3,198	1,913	6.58	274
Lisa	F	3,828	2,354	7.88	328
Stam	F	4,295	2,574	8.84	368
Nana	F	4,393	2,695	9.04	377
BraBrou*	M	2,189	2,189	7.40	308
Kigali*	M	2,430	2,430	8.21	342
Tonnerre*	M	2,504	2,504	8.46	352
Nze	M	3,750	2,296	7.72	322
David	M	3,773	2,447	7.76	323
Mambo	M	3,812	2,121	7.84	327
Mboumba	M	3,843	2,332	7.91	329
Wongo	M	4,020	2,353	8.27	345
Kengue	M	4,128	2,527	8.49	354
Mba	M	4,535	2,752	9.33	389
**Female Mean**		**3,367**	**2,326**	**7.87**	**328**
**Male Mean**		**3,498**	**2,395**	**8.14**	**339**
**Mean**		**3,444**	**2,367**	**8.03**	**334**

*Elephants with GPS transmission starting on May 12, 2016 instead of November 4, 2015 had fewer points and therefore shorter total distances. Total distance-10 month shows total distance over the 10 months of data available for all elephants (May 2016- March 2017). All other columns were calculated using all available data points for each elephant.
