# Supplementary material for: Forest elephant movement and habitat use in a tropical forest-grassland mosaic in Gabon
Source: PLoS One. 2018 Jul 11;13(7):e0199387. doi: 10.1371/journal.pone.0199387 (PMC6040693; doi:10.1371/journal.pone.0199387)
Supplement: S8 Table — (PDF) [file pone.0199387.s008.pdf]

S8 Table. Area of MCP home ranges. Home ranges expressed in square kilometers for different percentages of points after outlier removal (80 – 100% of all points). Table sorted by sex, then 95% MCP area.

		Area of MCP Home Range (km^2)				
**Elephant Name**	**Sex**	**80%**	**85%**	**90%**	**95%**	**100%**
Ndeka	F	46	64	90	122	286
Nana	F	97	115	141	174	239
Stam	F	133	142	155	177	314
Rosa	F	143	162	196	220	260
Lisa	F	162	185	233	308	447
Nongo	F	235	290	320	348	397
Malaika	F	358	381	407	430	534
Mba	M	111	132	170	229	427
Kigali	M	198	214	236	263	306
BraBrou	M	241	325	357	372	395
Wongo	M	291	317	365	492	731
Kengue	M	448	465	473	506	615
Mambo	M	221	324	434	533	723
Nze	M	191	482	615	819	1,425
Tonnere	M	683	780	858	932	1,030
David	M	1,077	1,356	1,436	1,467	1,745
Mboumba	M	1,502	1,634	1,910	2,047	2,253
**Female Mean**		**168**	**191**	**220**	**254**	**354**
**Male Mean**		**496**	**603**	**685**	**766**	**965**
**Mean**		**361**	**433**	**494**	**555**	**713**
