# Supplementary material for: Forest elephant movement and habitat use in a tropical forest-grassland mosaic in Gabon
Source: PLoS One. 2018 Jul 11;13(7):e0199387. doi: 10.1371/journal.pone.0199387 (PMC6040693; doi:10.1371/journal.pone.0199387)
Supplement: S9 Table — (PDF) [file pone.0199387.s009.pdf]

S9 Table. 100% MCP home range areas for WW by elephant and season.

Elephant Name	Sex	Dry MCP area (km^2)	Wet MCP area (km^2)
Ndeka	F	113	242
Rosa	F	177	259
Stam	F	188	312
Nana	F	205	193
Lisa	F	343	374
Nongo	F	392	138
Malaika	F	409	294
Nze	M	164	1,424
Kigali	M	306	199
Mba	M	333	163
BraBrou	M	359	96
Kengue	M	451	557
Mambo	M	632	501
Wongo	M	697	587
Tonnerre	M	811	384
David	M	1,425	1,512
Mboumba	M	1,946	1,918
**Female Mean**		**261**	**259**
**Male Mean**		**712**	**734**
**Mean**		**527**	**538**
