# Supplementary material for: Forest elephant movement and habitat use in a tropical forest-grassland mosaic in Gabon
Source: PLoS One. 2018 Jul 11;13(7):e0199387. doi: 10.1371/journal.pone.0199387 (PMC6040693; doi:10.1371/journal.pone.0199387)
Supplement: S10 Table — (PDF) [file pone.0199387.s010.pdf]

S10 Table. Area of KUD home ranges. Home ranges expressed in square kilometers for different probability levels of elephant utilization (80 – 95%). 50% KUDs represent core home range areas. Table sorted by sex, then 95% KUD area.

		Area of KUD Home Range (km^2)				
**Elephant Name**	**Sex**	50%	80%	85%	90%	95%
Ndeka	F	12	38	50	69	105
Nana	F	35	85	100	121	155
Stam	F	39	95	112	134	172
Rosa	F	47	115	133	156	192
Nongo	F	42	109	130	161	217
Lisa	F	50	128	155	194	254
Malaika	F	86	208	246	298	388
Mba	M	29	79	94	115	147
BraBrou	M	35	106	131	171	245
Kigali	M	64	148	171	201	247
Mambo	M	52	180	225	285	374
Wongo	M	75	183	222	280	388
Kengue	M	84	265	322	399	512
Nze	M	55	248	341	479	700
Tonnere	M	185	459	535	636	788
David	M	174	580	713	900	1,229
Mboumba	M	297	985	1,204	1,494	1,913
**Female Mean**		**44**	**111**	**132**	**162**	**212**
**Male Mean**		**105**	**323**	**396**	**496**	**654**
**Mean**		**80**	**236**	**287**	**358**	**472**
