# Supplementary material for: Forest elephant movement and habitat use in a tropical forest-grassland mosaic in Gabon
Source: PLoS One. 2018 Jul 11;13(7):e0199387. doi: 10.1371/journal.pone.0199387 (PMC6040693; doi:10.1371/journal.pone.0199387)
Supplement: S11 Table — (PDF) [file pone.0199387.s011.pdf]

S11 Table. Full model selection output for the factors influencing elephant movement in the dry season.

Where: ✓ = categorical terms included in the model; TOD = Time of day; df = Degrees of Freedom; AICc = Akaike's Information Criterion corrected for small sample size; W = model weight; AW = Adjusted weight across all models in the top set; grey shaded area = all models in the top model set (ΔAICc < 6 and non-nested).

Intercept	EVI	Road Distance	Sex	Slope	Stream Distance	Village Distance	df	AICc	ΔAICc	W	AW
0.011	0.129			0.046	0.060	-0.263	6	9866.2	0.00	0.31	0.61
0.011	0.133				0.060	-0.260	5	9868.0	1.73	0.13	0.26
0.044	0.130		✓	0.046	0.060	-0.263	7	9868.0	1.78	0.13	-
0.011	0.130	-0.004		0.046	0.060	-0.264	7	9868.2	1.99	0.12	-
0.045	0.133		✓		0.059	-0.260	6	9869.7	3.49	0.06	-
0.011	0.135	-0.010			0.060	-0.261	6	9869.8	3.58	0.05	-
0.043	0.130	-0.003	✓	0.046	0.060	-0.263	8	9870.0	3.77	0.05	-
0.011	0.129			0.046		-0.257	5	9870.0	3.80	0.05	0.09
0.044	0.136	-0.010	✓		0.060	-0.261	7	9871.6	5.35	0.02	-
0.011	0.133					-0.254	4	9871.7	5.49	0.02	0.04
0.046	0.130		✓	0.046		-0.256	6	9871.8	5.53	0.02	-
0.011	0.130	-0.003		0.046		-0.257	6	9872.0	5.79	0.02	-
0.047	0.133		✓			-0.253	5	9873.4	7.20	0.01	-
0.011	0.135	-0.010				-0.255	5	9873.6	7.35	0.01	-
0.045	0.131	-0.003	✓	0.046		-0.256	7	9873.8	7.53	0.01	-
0.046	0.136	-0.009	✓			-0.254	6	9875.3	9.07	0.00	-
0.010				0.055	0.060	-0.296	5	9889.8	23.52	0.00	-
0.010		0.032		0.058	0.060	-0.291	6	9890.2	23.95	0.00	-
0.030			✓	0.055	0.060	-0.296	6	9891.7	25.44	0.00	-
0.033		0.032	✓	0.058	0.060	-0.291	7	9892.1	25.83	0.00	-
0.010					0.060	-0.294	4	9893.0	26.81	0.00	-
0.010				0.055		-0.289	4	9893.7	27.43	0.00	-
0.010		0.025			0.060	-0.289	5	9894.1	27.82	0.00	-
0.010		0.032		0.058		-0.284	5	9894.1	27.82	0.00	-
0.031			✓		0.060	-0.293	5	9895.0	28.72	0.00	-
0.032			✓	0.055		-0.289	5	9895.6	29.32	0.00	-
0.035		0.032	✓	0.058		-0.284	6	9895.9	29.67	0.00	-
0.034		0.025	✓		0.060	-0.289	6	9895.9	29.70	0.00	-
0.010						-0.286	3	9896.9	30.67	0.00	-
0.010		0.025				-0.282	4	9897.9	31.65	0.00	-
0.033			✓			-0.286	4	9898.8	32.55	0.00	-
0.004	0.151			0.040	0.052		5	9899.2	32.95	0.00	-
0.036		0.026	✓			-0.282	5	9899.7	33.49	0.00	-
0.004	0.153				0.052		4	9900.0	33.79	0.00	-

0.026	0.153		✓	0.039	0.051	6	9900.6	34.33	0.00	-
0.004	0.151	0.002		0.040	0.052	6	9901.2	34.95	0.00	-
0.028	0.155		✓		0.051	5	9901.3	35.09	0.00	-
0.004	0.151			0.040		4	9902.0	35.76	0.00	-
0.004	0.154	-0.004			0.052	5	9902.0	35.77	0.00	-
0.027	0.152	0.004	✓	0.040	0.051	7	9902.6	36.32	0.00	-
0.004	0.153					3	9902.8	36.60	0.00	-
0.029	0.153		✓	0.039		5	9903.2	37.01	0.00	-
0.028	0.156	-0.002	✓		0.051	6	9903.3	37.09	0.00	-
0.030	0.155		✓			4	9904.0	37.74	0.00	-
0.004	0.151	0.001		0.040		5	9904.0	37.77	0.00	-
0.004	0.154	-0.005				4	9904.8	38.56	0.00	-
0.029	0.152	0.003	✓	0.040		6	9905.2	39.00	0.00	-
0.030	0.156	-0.003	✓			5	9906.0	39.73	0.00	-
0.004		0.046		0.053	0.052	5	9936.0	69.75	0.00	-
0.004				0.047	0.051	4	9937.7	71.46	0.00	-
0.016		0.047	✓	0.053	0.052	6	9937.8	71.58	0.00	-
0.004		0.045		0.053		4	9938.8	72.59	0.00	-
0.004		0.040			0.052	4	9938.9	72.66	0.00	-
0.010			✓	0.047	0.051	5	9939.6	73.41	0.00	-
0.004					0.051	3	9939.7	73.45	0.00	-
0.004				0.047		3	9940.3	74.10	0.00	-
0.018		0.046	✓	0.052		5	9940.6	74.34	0.00	-
0.017		0.041	✓		0.051	5	9940.7	74.45	0.00	-
0.012			✓		0.051	4	9941.6	75.38	0.00	-
0.004		0.038				3	9941.7	75.45	0.00	-
0.013			✓	0.047		4	9942.2	76.01	0.00	-
0.004						2	9942.3	76.08	0.00	-
0.019		0.040	✓			4	9943.4	77.17	0.00	-
0.014			✓			3	9944.2	77.95	0.00	-
