# Supplementary material for: Forest elephant movement and habitat use in a tropical forest-grassland mosaic in Gabon
Source: PLoS One. 2018 Jul 11;13(7):e0199387. doi: 10.1371/journal.pone.0199387 (PMC6040693; doi:10.1371/journal.pone.0199387)
Supplement: S12 Table — (PDF) [file pone.0199387.s012.pdf]

**S12 Table. Full model selection output for the factors influencing elephant movement in the wet season.**

Where: ✓ = categorical terms included in the model; TOD = Time of day; df = Degrees of Freedom; AICc = Akaike's Information Criterion corrected for small sample size; W = model weight; AW = Adjusted weight across all models in the top set; grey shaded area = all models in the top model set (ΔAICc < 6 and non-nested).

| Intercept | EVI | Road Distance | Sex | Slope | Stream Distance | Village Distance | df | AICc | ΔAICc | W | AW |
|---|---|---|---|---|---|---|---|---|---|---|---|
| 0.016 | 0.221 | -0.090 | | | 0.185 | -0.083 | 6 | 10016.3 | 0.00 | 0.44 | 0.85 |
| 0.034 | 0.222 | -0.090 | ✓ | | 0.184 | -0.083 | 7 | 10018.1 | 1.79 | 0.18 | - |
| 0.016 | 0.220 | -0.090 | | 0.006 | 0.185 | -0.084 | 7 | 10018.3 | 1.95 | 0.17 | - |
| 0.013 | 0.232 | -0.089 | | | 0.181 | | 5 | 10019.8 | 3.50 | 0.08 | 0.15 |
| 0.034 | 0.221 | -0.090 | ✓ | 0.006 | 0.184 | -0.083 | 8 | 10020.0 | 3.74 | 0.07 | - |
| 0.030 | 0.233 | -0.089 | ✓ | | 0.180 | | 6 | 10021.5 | 5.16 | 0.03 | - |
| 0.013 | 0.231 | -0.089 | | 0.003 | 0.181 | | 6 | 10021.8 | 5.49 | 0.03 | - |
| 0.030 | 0.232 | -0.088 | ✓ | 0.003 | 0.180 | | 7 | 10023.5 | 7.15 | 0.01 | - |
| 0.018 | 0.208 | | | | 0.187 | -0.087 | 5 | 10027.4 | 11.08 | 0.00 | - |
| 0.018 | 0.206 | | | 0.012 | 0.186 | -0.088 | 6 | 10029.1 | 12.85 | 0.00 | - |
| 0.039 | 0.208 | | ✓ | | 0.186 | -0.086 | 6 | 10029.2 | 12.86 | 0.00 | - |
| 0.039 | 0.207 | | ✓ | 0.012 | 0.186 | -0.087 | 7 | 10030.9 | 14.61 | 0.00 | - |
| 0.014 | 0.216 | | | | 0.183 | | 4 | 10031.2 | 14.89 | 0.00 | - |
| 0.034 | 0.217 | | ✓ | | 0.182 | | 5 | 10032.7 | 16.43 | 0.00 | - |
| 0.014 | 0.215 | | | 0.009 | 0.183 | | 5 | 10033.0 | 16.73 | 0.00 | - |
| 0.034 | 0.216 | | ✓ | 0.010 | 0.182 | | 6 | 10034.6 | 18.27 | 0.00 | - |
| 0.013 | 0.210 | -0.095 | | | | -0.072 | 5 | 10072.3 | 55.95 | 0.00 | - |
| 0.041 | 0.211 | -0.095 | ✓ | | | -0.066 | 6 | 10073.4 | 57.12 | 0.00 | - |
| 0.013 | 0.209 | -0.094 | | 0.011 | | -0.073 | 6 | 10074.0 | 57.74 | 0.00 | - |
| 0.041 | 0.210 | -0.094 | ✓ | 0.011 | | -0.066 | 7 | 10075.2 | 58.90 | 0.00 | - |
| 0.012 | 0.227 | -0.091 | | | | | 4 | 10075.7 | 59.41 | 0.00 | - |
| 0.043 | 0.229 | -0.090 | ✓ | | | | 5 | 10076.6 | 60.26 | 0.00 | - |
| 0.012 | 0.227 | -0.091 | | 0.007 | | | 5 | 10077.6 | 61.32 | 0.00 | - |
| 0.043 | 0.229 | -0.090 | ✓ | 0.007 | | | 6 | 10078.5 | 62.17 | 0.00 | - |
| 0.016 | 0.197 | | | | | -0.086 | 4 | 10084.7 | 68.37 | 0.00 | - |
| 0.048 | 0.198 | | ✓ | | | -0.081 | 5 | 10086.0 | 69.65 | 0.00 | - |
| 0.016 | 0.195 | | | 0.017 | | -0.087 | 5 | 10086.2 | 69.85 | 0.00 | - |
| 0.014 | | -0.059 | | | 0.173 | -0.133 | 5 | 10086.4 | 70.11 | 0.00 | - |
| 0.014 | | -0.057 | | 0.026 | 0.172 | -0.135 | 6 | 10087.2 | 70.88 | 0.00 | - |
| 0.048 | 0.196 | | ✓ | 0.017 | | -0.082 | 6 | 10087.4 | 71.12 | 0.00 | - |
| 0.012 | 0.207 | | | | | | 3 | 10088.1 | 71.76 | 0.00 | - |
| 0.011 | | -0.059 | ✓ | | 0.173 | -0.133 | 6 | 10088.4 | 72.11 | 0.00 | - |
| 0.046 | 0.210 | | ✓ | | | | 4 | 10088.6 | 72.29 | 0.00 | - |

| | | | | | | | | | | | |
|---|---|---|---|---|---|---|---|---|---|---|---|
| 0.012 | | -0.057 | ✓ | 0.026 | 0.172 | -0.134 | 7 | 10089.2 | 72.88 | 0.00 | - |
| 0.012 | 0.207 | | | 0.015 | | | 4 | 10089.7 | 73.36 | 0.00 | - |
| 0.046 | 0.209 | | ✓ | 0.015 | | | 5 | 10090.2 | 73.89 | 0.00 | - |
| 0.014 | | | | | 0.175 | -0.133 | 4 | 10090.3 | 73.99 | 0.00 | - |
| 0.014 | | | | 0.030 | 0.174 | -0.134 | 5 | 10090.7 | 74.42 | 0.00 | - |
| 0.016 | | | ✓ | | 0.175 | -0.133 | 5 | 10092.3 | 75.99 | 0.00 | - |
| 0.016 | | | ✓ | 0.030 | 0.174 | -0.134 | 6 | 10092.7 | 76.42 | 0.00 | - |
| 0.013 | | -0.041 | | | 0.175 | | 4 | 10107.6 | 91.26 | 0.00 | - |
| 0.013 | | | | | 0.176 | | 3 | 10108.5 | 92.24 | 0.00 | - |
| 0.013 | | -0.039 | | 0.021 | 0.175 | | 5 | 10108.8 | 92.47 | 0.00 | - |
| 0.013 | | | | 0.024 | 0.175 | | 4 | 10109.5 | 93.22 | 0.00 | - |
| 0.010 | | -0.041 | ✓ | | 0.175 | | 5 | 10109.6 | 93.25 | 0.00 | - |
| 0.012 | | | ✓ | | 0.176 | | 4 | 10110.5 | 94.25 | 0.00 | - |
| 0.010 | | -0.039 | ✓ | 0.021 | 0.175 | | 6 | 10110.8 | 94.46 | 0.00 | - |
| 0.013 | | | ✓ | 0.024 | 0.175 | | 5 | 10111.5 | 95.23 | 0.00 | - |
| 0.013 | | -0.065 | | | | -0.123 | 4 | 10136.2 | 119.93 | 0.00 | - |
| 0.013 | | -0.063 | | 0.030 | | -0.125 | 5 | 10136.6 | 120.28 | 0.00 | - |
| 0.021 | | -0.064 | ✓ | | | -0.123 | 5 | 10138.1 | 121.83 | 0.00 | - |
| 0.022 | | -0.062 | ✓ | 0.030 | | -0.124 | 6 | 10138.5 | 122.18 | 0.00 | - |
| 0.013 | | | | 0.034 | | -0.120 | 4 | 10141.5 | 125.18 | 0.00 | - |
| 0.013 | | | | | | -0.121 | 3 | 10141.6 | 125.27 | 0.00 | - |
| 0.026 | | | ✓ | 0.034 | | -0.119 | 5 | 10143.3 | 126.97 | 0.00 | - |
| 0.026 | | | ✓ | | | -0.119 | 4 | 10143.4 | 127.08 | 0.00 | - |
| 0.013 | | -0.044 | | | | | 3 | 10161.0 | 144.71 | 0.00 | - |
| 0.013 | | -0.042 | | 0.025 | | | 4 | 10161.9 | 145.59 | 0.00 | - |
| 0.013 | | | | | | | 2 | 10162.5 | 146.17 | 0.00 | - |
| 0.023 | | -0.043 | ✓ | | | | 4 | 10162.9 | 146.57 | 0.00 | - |
| 0.013 | | | | 0.028 | | | 3 | 10163.1 | 146.76 | 0.00 | - |
| 0.023 | | -0.041 | ✓ | 0.025 | | | 5 | 10163.8 | 147.45 | 0.00 | - |
| 0.026 | | | ✓ | | | | 3 | 10164.3 | 147.95 | 0.00 | - |
| 0.026 | | | ✓ | 0.028 | | | 4 | 10164.8 | 148.54 | 0.00 | - |
