# Supplementary material for: Forest elephant movement and habitat use in a tropical forest-grassland mosaic in Gabon
Source: PLoS One. 2018 Jul 11;13(7):e0199387. doi: 10.1371/journal.pone.0199387 (PMC6040693; doi:10.1371/journal.pone.0199387)
Supplement: S1 Fig — (PDF) [file pone.0199387.s013.pdf]

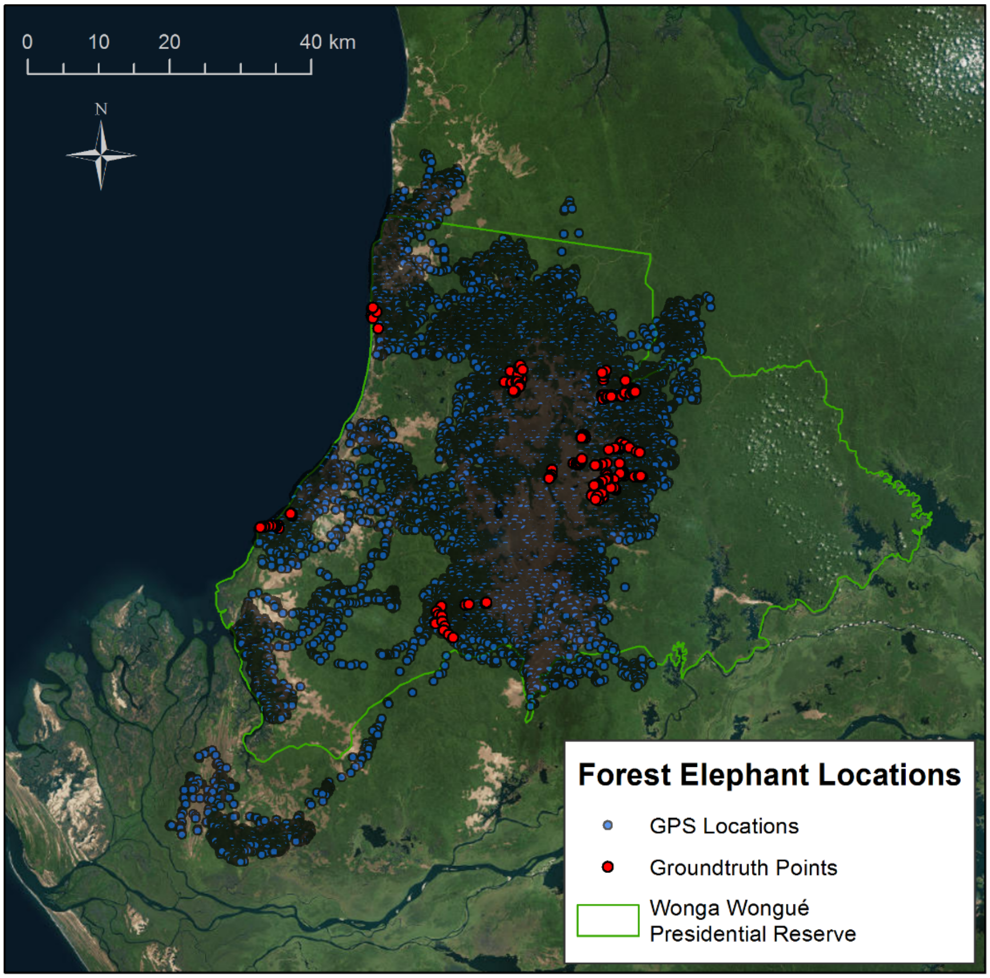

S1 Fig. GPS locations and ground truth points. GPS locations of 17 elephants (blue) in Wonga Wongué Presidential Reserve and 220 ground truth points (red) collected between June and July 2016.
