# Supplementary figures and images for: Forest elephant movement and habitat use in a tropical forest-grassland mosaic in Gabon
Source: PLoS One. 2018 Jul 11;13(7):e0199387. doi: 10.1371/journal.pone.0199387 (PMC6040693; doi:10.1371/journal.pone.0199387)

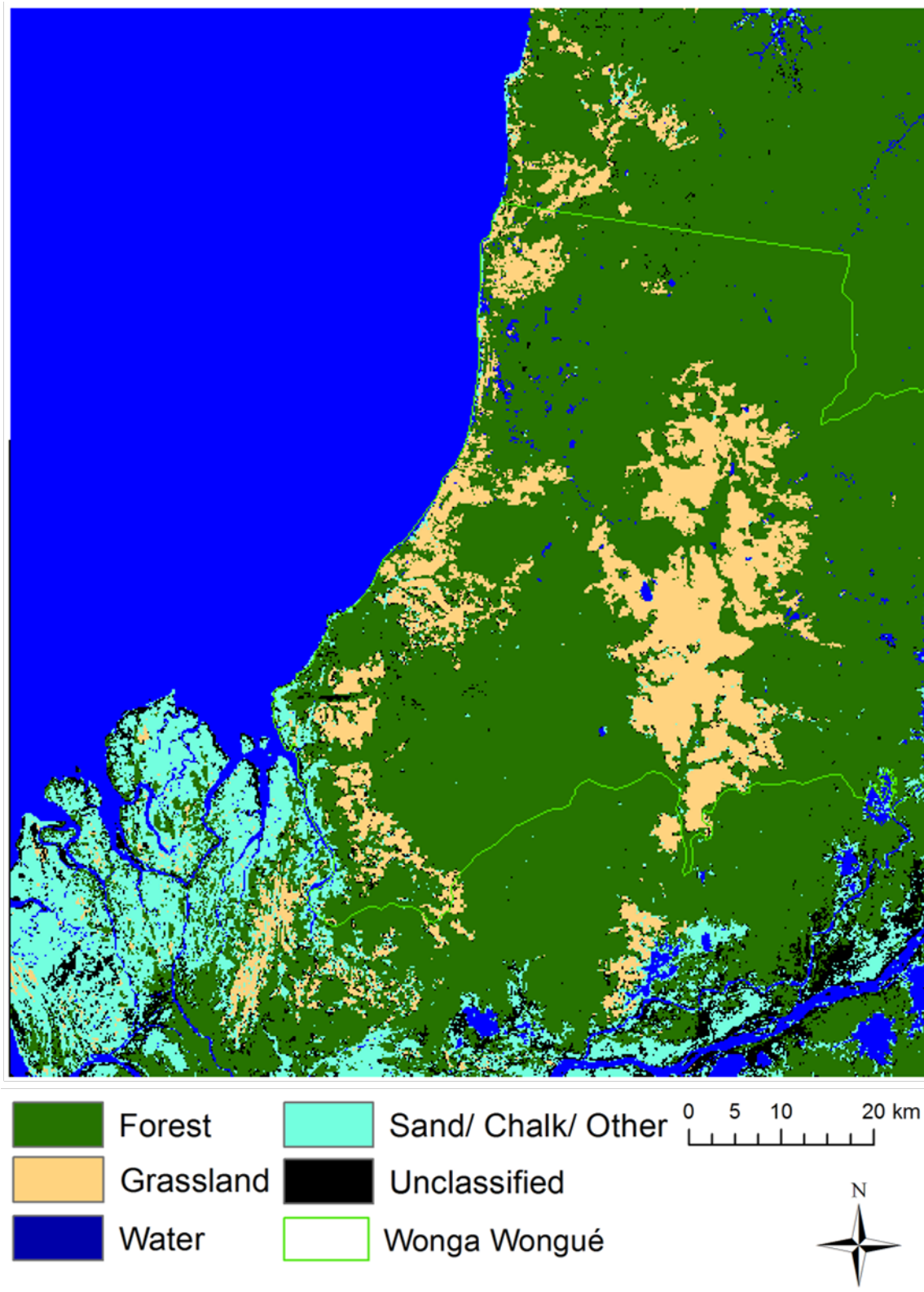

S2 Fig. Thematic land cover map for the study area.

Supplement: S2 Fig — (PDF) [file pone.0199387.s014.pdf]

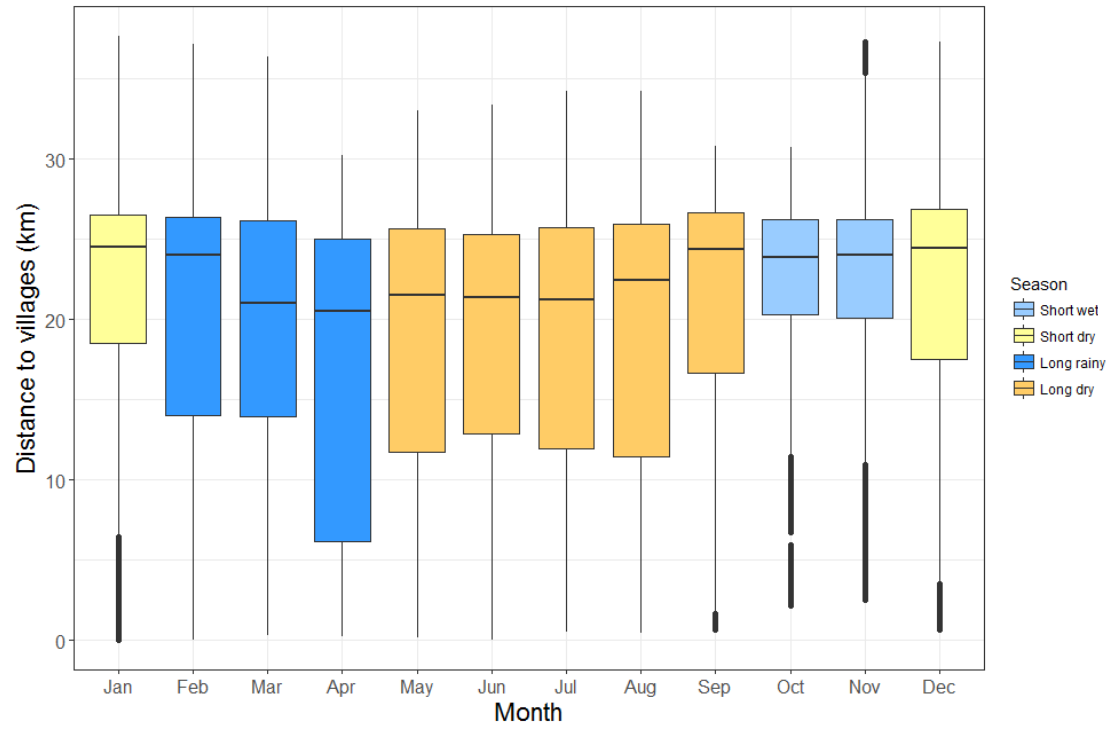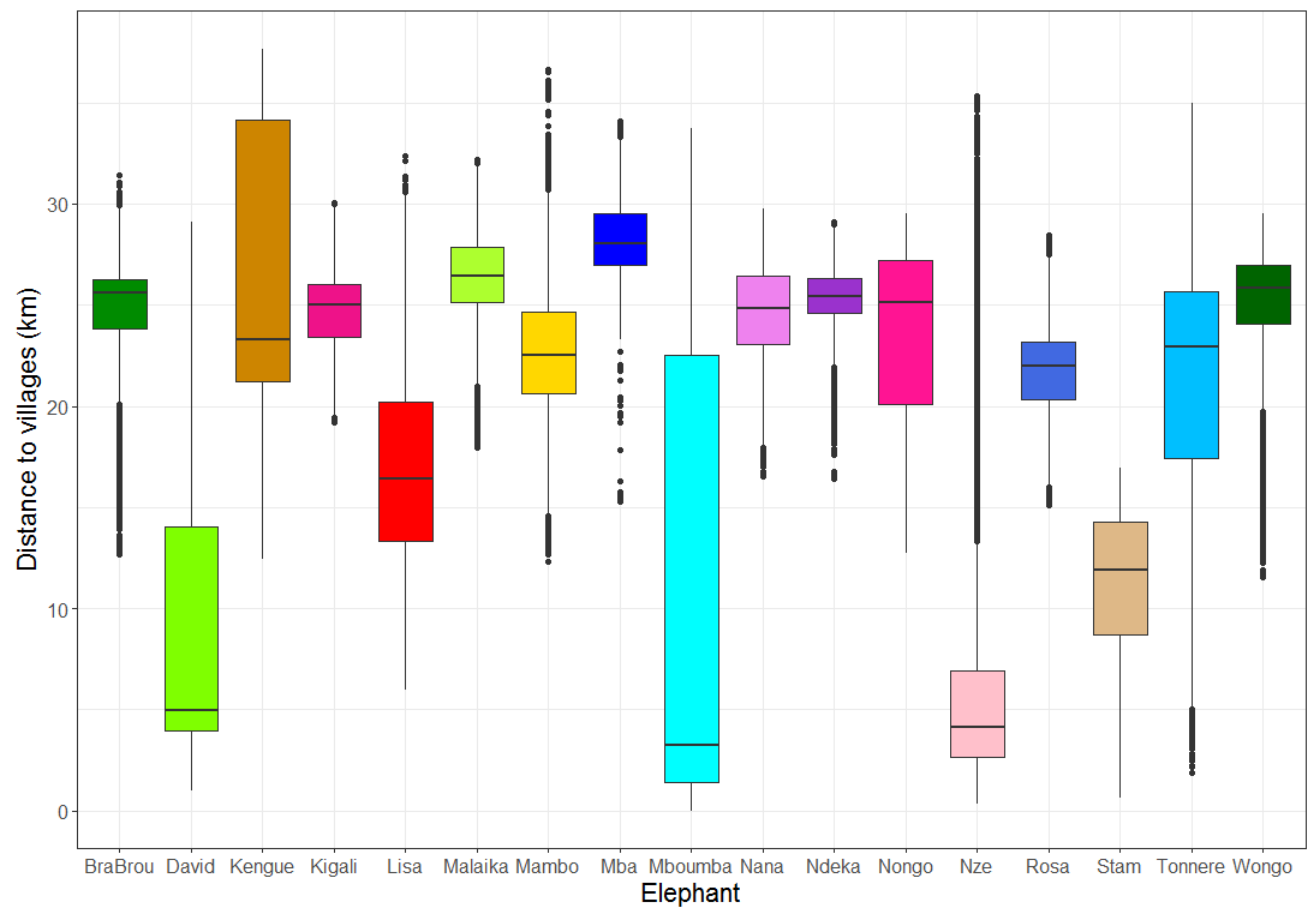

**S7 Fig. Distance to nearest villages by month (*above*) and by elephant (*below*).**

Supplement: S7 Fig — (PDF) [file pone.0199387.s019.pdf]
