# Supplementary material for: Forest elephant movement and habitat use in a tropical forest-grassland mosaic in Gabon
Source: PLoS One. 2018 Jul 11;13(7):e0199387. doi: 10.1371/journal.pone.0199387 (PMC6040693; doi:10.1371/journal.pone.0199387)
Supplement: S3 Fig — (PDF) [file pone.0199387.s015.pdf]

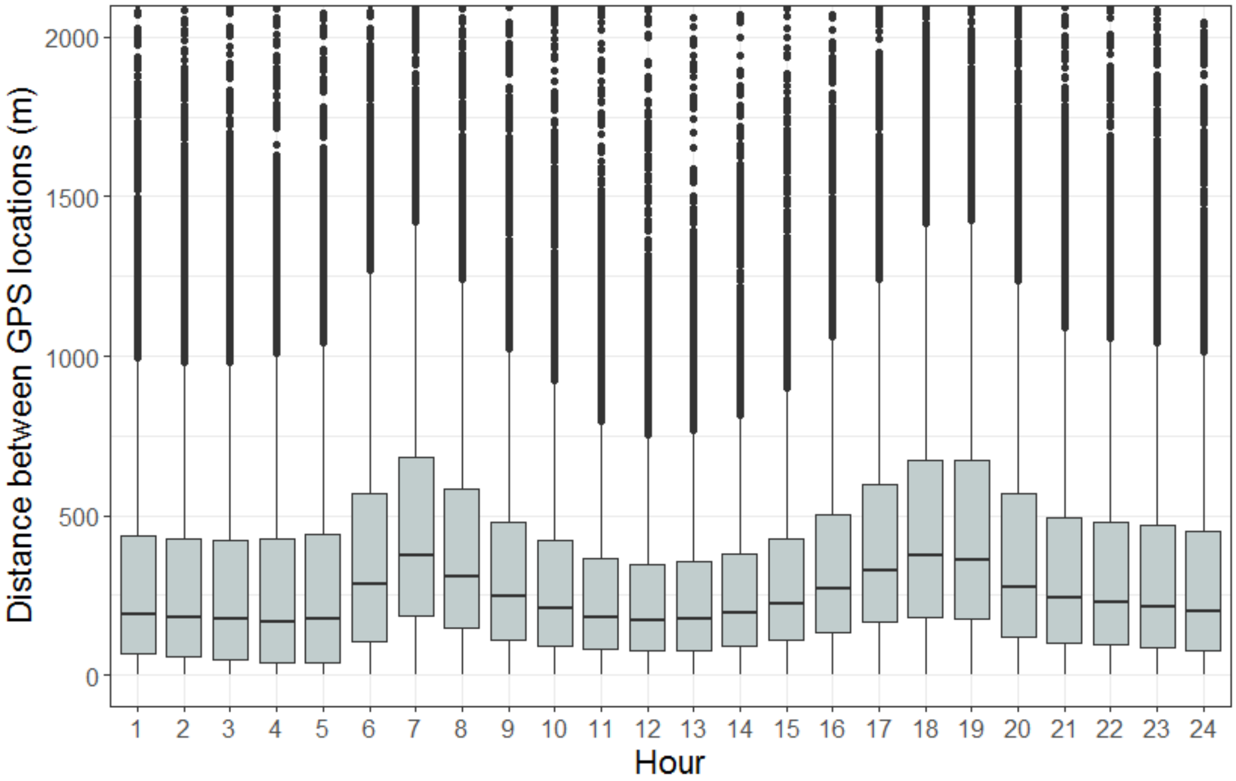

S3 Fig. Boxplots showing distances moved between consecutive GPS points for all hours of the day. Elephants exhibited peak movement times between 6:00 and 9:00 and between 17:00 and 20:00. The y-axis was limited to 2000 m to better illustrate the boxplots, rather than the outliers.
