# Supplementary material for: Forest elephant movement and habitat use in a tropical forest-grassland mosaic in Gabon
Source: PLoS One. 2018 Jul 11;13(7):e0199387. doi: 10.1371/journal.pone.0199387 (PMC6040693; doi:10.1371/journal.pone.0199387)
Supplement: S4 Fig — (PDF) [file pone.0199387.s016.pdf]

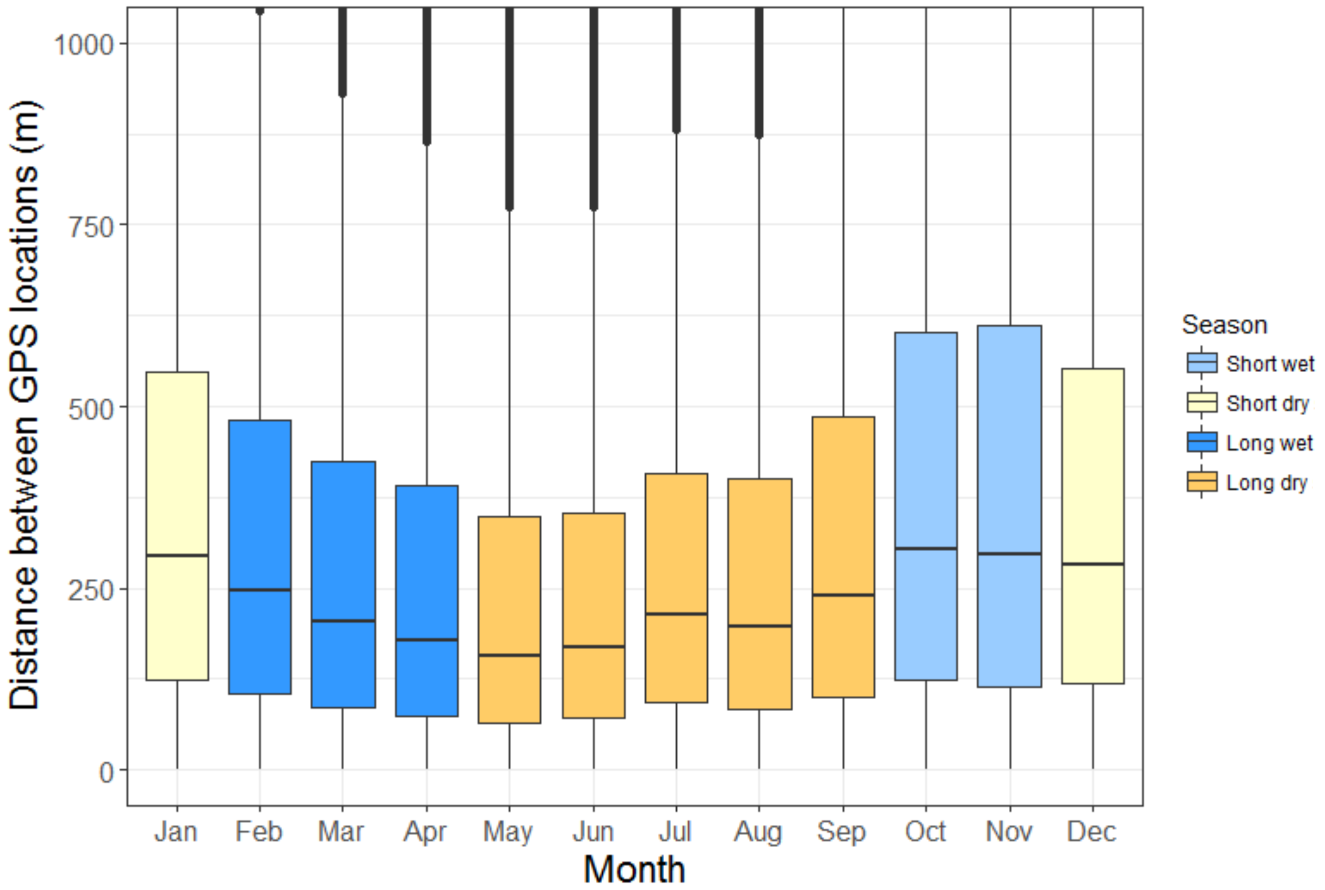

S4 Fig. Boxplots showing distributions of distance between consecutive GPS points by month. Elephants exhibited greatest movement speeds during the short-wet season (Oct-Nov mean = 448 m, range = 0-7.3 km. Elephants had the second longest hourly movements during the short-dry season of December-January (mean = 429 m, range: 0-8.0 km), followed by the long-wet season of February-April (mean = 350 m, range = 0-16.8 km), and the long-dry season (mean = 313 m, range = 0-8.1 km). The y-axis was limited to 1000 m to better illustrate the boxplots, rather than the outliers.
