# Supplementary material for: Forest elephant movement and habitat use in a tropical forest-grassland mosaic in Gabon
Source: PLoS One. 2018 Jul 11;13(7):e0199387. doi: 10.1371/journal.pone.0199387 (PMC6040693; doi:10.1371/journal.pone.0199387)
Supplement: S5 Fig — (PDF) [file pone.0199387.s017.pdf]

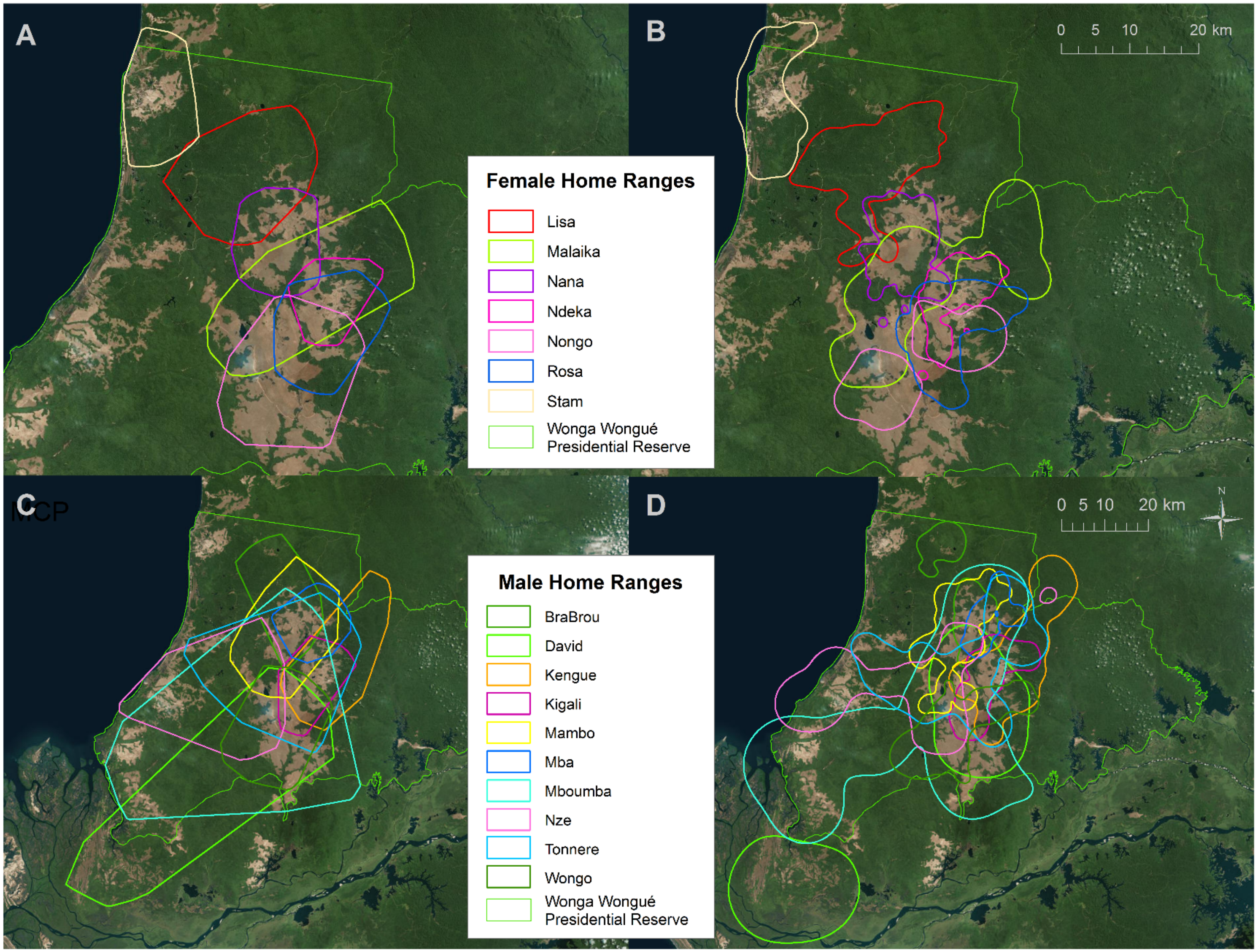

S5 Fig. 95% MCP and 95% KUD home ranges for forest elephants in WW. (A) MCP home ranges for females, (B) KUD home ranges for females, (C) MCP home ranges for males, and (D) KUD home ranges for males.
