# Supplementary material for: Forest elephant movement and habitat use in a tropical forest-grassland mosaic in Gabon
Source: PLoS One. 2018 Jul 11;13(7):e0199387. doi: 10.1371/journal.pone.0199387 (PMC6040693; doi:10.1371/journal.pone.0199387)
Supplement: S6 Fig — (PDF) [file pone.0199387.s018.pdf]

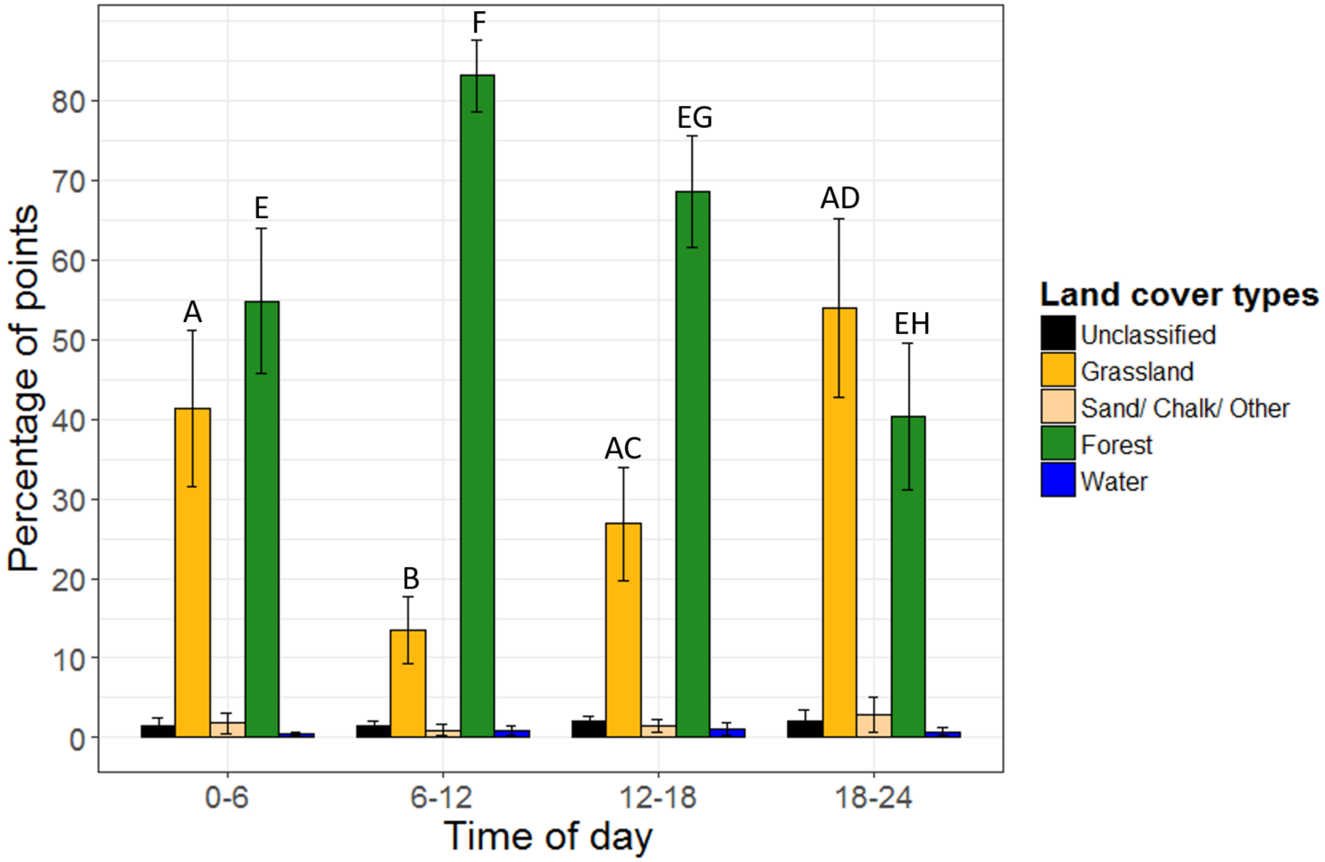

S6 Fig. Percentage of elephant locations in each land cover type by time of day. Pairwise comparisons with different letters indicate significant differences in proportion of use by elephants across time periods within land cover type. Error bars represent 95% confidence intervals. Elephants spent more time in the grassland during late evening hours than daylight hours.
