# Supplementary material for: Forest elephant movement and habitat use in a tropical forest-grassland mosaic in Gabon
Source: PLoS One. 2018 Jul 11;13(7):e0199387. doi: 10.1371/journal.pone.0199387 (PMC6040693; doi:10.1371/journal.pone.0199387)
Supplement: S1 Text — (PDF) [file pone.0199387.s020.pdf]

S1 Text. Land cover classification: Detailed protocol.

We obtained Landsat 8 OLI/TIRS imagery (30 m resolution; S2 Table) from the U.S. Geological Survey (USGS) [24]. At the L1-T processing level, the imagery tiles were radiometrically, geometrically, and terrain-corrected. We used four images: one pair of images from June 28, 2015 during the long-dry season, and a second pair of images from March 10, 2016 during the long-wet season (S2 Table). Images were selected based on limited availability of cloud-free images and best temporal overlap with the middle of each season. Two tiles were sufficient to cover the study area, each with a swath width of 185 km.

All remote sensing analyses were performed in ENVI version 5.3 [25], unless otherwise specified. We calibrated the images to top-of-atmosphere reflectance, and then conducted atmospheric corrections on all tiles for Bands 1-4 via a dark object subtraction method, as recommended by Song *et al.*[1] We chose the subtracted value to be the band minimum for each band.

To ground truth the remote sensing data, ECM collected habitat and vegetation data in WW from May-July 2016, characterizing 220 recent elephant locations on attributes including land cover type, canopy cover, understory density, tree height, and most abundant tree species (S1 Fig, S3 Table). Sampling of ground truth points was stratified across elephant sex, geographical area, and general land cover (e.g., grassland vs. beach, vs. forest).

We created a thematic land cover map for the study area using the pair of images taken during the dry season because the ground truth data was collected during the dry season. To distinguish land cover categories, we employed maximum likelihood supervised classification using regions of interest (ROIs) created from the ground truth points as a training sample. The

---

[1] Song C, Woodcock CE, Seto KC, Lenney MP, Macomber SA. Classification and change detection using Landsat TM data: when and how to correct atmospheric effects? Remote Sens Environ. 2001;75:230-244.

four main cover types of interest were Forest, Grassland, Water, and Sand/Chalk/Other. We excluded ground truth points in swamps and mangroves because collared elephants rarely ventured into these land cover types and they were not easily distinguishable from forest (see S3 Table for final ROIs). After ensuring high ROI separability (Jeffries-Matusita), we conducted maximum likelihood supervised classification on each of the dry season tiles separately, using multiple value thresholds to reflect the scarcity of rare cover types and to minimize unclassified pixels: 0.0001 for Sand/Other, 0.001 for Water, Grassland, and Forest.

Post-processing, we used sieving to remove isolated pixels and clumping to group adjacent similarly classified pixels, combining the two classifications together to produce the final thematic land cover map in ArcMap version 10.4.1 [31]. We performed an accuracy assessment on the final classification using randomly generated ground truth points and Google Earth to evaluate the performance of the supervised classification technique. We excluded unclassified and rare pixels (those containing sand, chalk or other habitat) because we were primarily interested in correctly classifying forest vs. grassland types.

For all tiles, we created spectral enhancement bands for enhanced vegetation index (EVI), using coefficients adopted in the MODIS-EVI algorithm [24], and Tasseled Cap Transformation (TCT) bands for Brightness, Greenness, and Wetness, using coefficients derived by Baig et al. [26]. We generated these rasters for later use as habitat covariates in habitat modeling (See *Drivers of Elephant Movements* methods).
